# Supplementary material for: Absent thermal equilibration on fractional quantum Hall edges over macroscopic scale
Source: Nat Commun. 2022 Jan 19;13:376. doi: 10.1038/s41467-022-28009-0 (PMC8770503; doi:10.1038/s41467-022-28009-0)
Supplement: Supplementary file 1 — Supplementary information [file 41467_2022_28009_MOESM1_ESM.pdf]

Supplementary Information for "Absent thermal equilibration on fractional quantum Hall edges over macroscopic scale"

Ron Aharon Melcer,[1] Bivas Dutta,[1] Christian Spånslätt,[2,3,4]
Jinhong Park,[5] Alexander D. Mirlin,[3,4,6,7] and Vladimir Umansky[1]

[1] *Braun Center for Submicron Research, Department of Condensed Matter Physics, Weizmann Institute of Science, Rehovot 761001, Israel*
[2] *Department of Microtechnology and Nanoscience (MC2), Chalmers University of Technology, S-412 96 Göteborg, Sweden*
[3] *Institute for Quantum Materials and Technologies, Karlsruhe Institute of Technology, 76021 Karlsruhe, Germany*
[4] *Institut für Theorie der Kondensierten Materie, Karlsruhe Institute of Technology, 76128 Karlsruhe, Germany*
[5] *Institute for Theoretical Physics, University of Cologne, Zülpicher Str. 77, 50937 Köln, Germany*
[6] *Petersburg Nuclear Physics Institute, 188300 St. Petersburg, Russia*
[7] *L. D. Landau Institute for Theoretical Physics RAS, 119334 Moscow, Russia*

(Dated: November 10, 2021)

Supplementary Note 1. TECHNICAL DETAILS OF NOISE MEASUREMENTS

As described in the main text, upstream and downstream noise was measured in two distinct amplifiers. Both amplifiers are connected in parallel to an RLC circuit. In our case the inductance comes from a superconducting coil located at the mixing chamber plate, the capacitance is the line capacitance leading from the sample to the homemade cryo-amplifier located at the $4.2K$ plate, and $R = G_{2T}^{-1} = h/(\nu e^2)$ is the sample resistance. We configured the inductance of the coils such that the resonance curves of the two amplifiers don't overlap: the central frequencies are f_D = 693kHz and f_U = 633kHz (with indices D and U denoting "downstream" and "upstream", respectively). The bandwidth of both circuits is 15kHz (14kHz) for $\nu = 2/3$ ($\nu = 3/5$). In this case, in the frequency range being

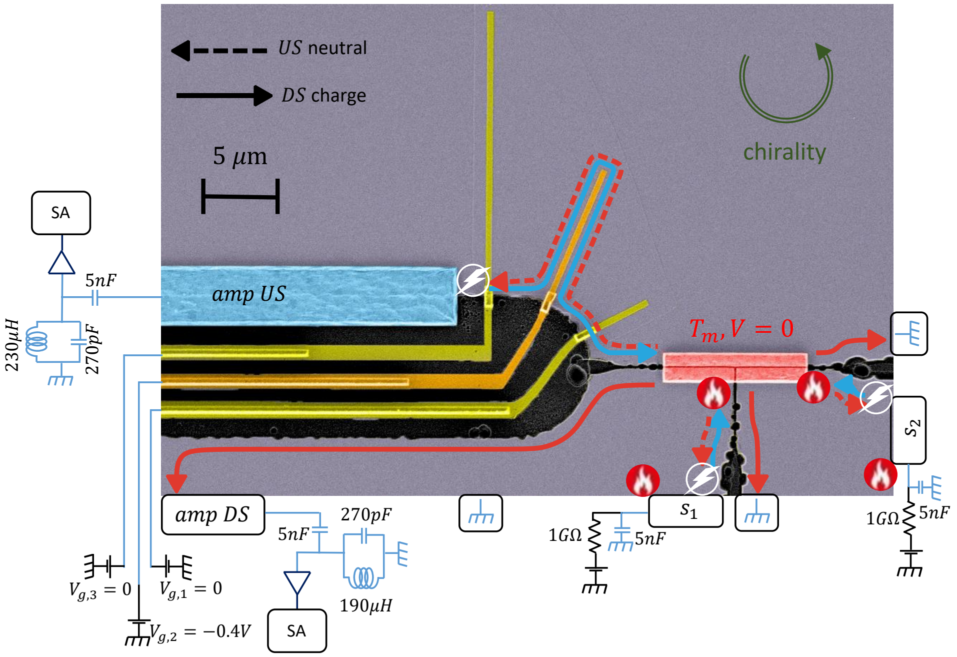

Supplementary Figure 1. **Device image.** False color SEM image of device B1, similar to Fig. 3a in the main text. Here, we have denoted all hot spots and noise spots that form in the experiment. In addition, we have specified the circuit components used in our measurements: the color of a component corresponds to its temperature: room temperature (black), 4.2K (dark blue) and 6mK (light blue).

picked up by the downstream amplifier, the upstream amplifier contact is simply a short to ground (see Supplementary Figure 1 for a detailed description of the measurement circuit).

This configuration simplifies the analysis of the downstream noise, which is used for extraction of the temperature T_m of the central floating island Ω_m. This is described in more detail in Supplementary Note 12. The measured noise in the downstream amplifier includes several contributions. The major contribution comes from Johnson-Nyquist noise generated at the central floating contact due to its elevated temperature. A second contribution comes from reflected upstream noise. In the frequency bandwidth where the downstream noise is measured, both the upstream amplifier contact and the source contacts S_1 and S_2 are effectively grounded (the source contacts are connected via a 5nF capacitance to ground). Therefore, the current noise generated at the noise spots, next to these contacts, flows to Ω_m, and from there to the downstream amplifier. For more details, see Supplementary Note 6 B 1 below.

Supplementary Note 2. AMPLIFIER GAIN AND TEMPERATURE CALIBRATION

The method used to calibrate the amplifier gain and electron temperature is based on Johnson-Nyquist noise[1,2]: $S_V = 4A^2 k_B T / G_{2T}$, where A is the gain of the amplifiers, k_B is the Boltzmann constant, and T is the temperature. Linearly fitting the noise with the fridge temperature (see Supplementary Figure 2), allows us to extract the gain A and the base noise of the amplifiers S_{base}. The base noise is used to extract the temperature T_0 of the electrons. At the lowest temperature (typically below 20mK) the electron temperature can be higher than that of the cryostat. We extract T_0 from: $T_0 = (S_V - S_{\mathrm{base}}) G_{2T} / (4A^2 k_B)$. In our measurements the cryostat temperature was always 6mK and T_0 was measured between 11mK and 14mK for all considered Hall states. An interesting observation is that T_0 tends to be smaller for states with larger κ_{2T}, such as $\nu = 3$ and $\nu = 3/5$, and a somewhat larger for states with small κ_{2T} such as $\nu = 1$ and $\nu = 2/3$. This observation suggests that at low temperatures, the most efficient cooling mechanism of the devices is via the edge modes.

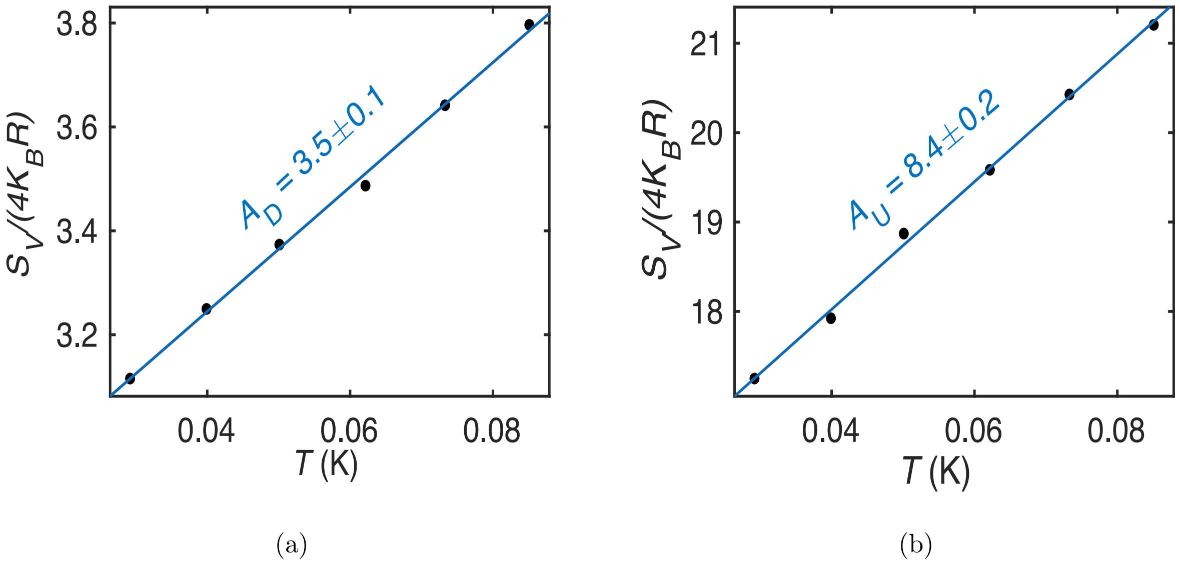

Supplementary Figure 2. **Amplifier calibration.** Voltage noise strength S_V as a function of the mixing chamber temperature T for (a) the downstream amplifier and (b) the upstream amplifier. The slope of the linear fit is the gain squared (A^2).

Supplementary Note 3. THERMAL CONDUCTANCE MEASUREMENT OF INTEGER STATES

To check that the thermal conductance measurements work properly in our B-devices (depicted in Supplementary Figure 1), we performed thermal conductance measurements at the three integer fillings $\nu = 1, 2, 3$ (see Supplementary Figure 3). The measurement scheme was identical to the fractional states and is described in the main text (though for the integer states, all length-controlling gates were not operational, thereby fixing L to the smallest available value). We find good agreement with the expected quantizations $\kappa_{2T}/\kappa_0 = n_d$. For the integer states, where there are only downstream modes, neither equilibration nor dissipation influences the thermal conductance. The suppression of a single quantum of thermal conductance due to heat Coulomb blockade[3,4] was not observed in our devices due to the relatively large central Ohmic contact.

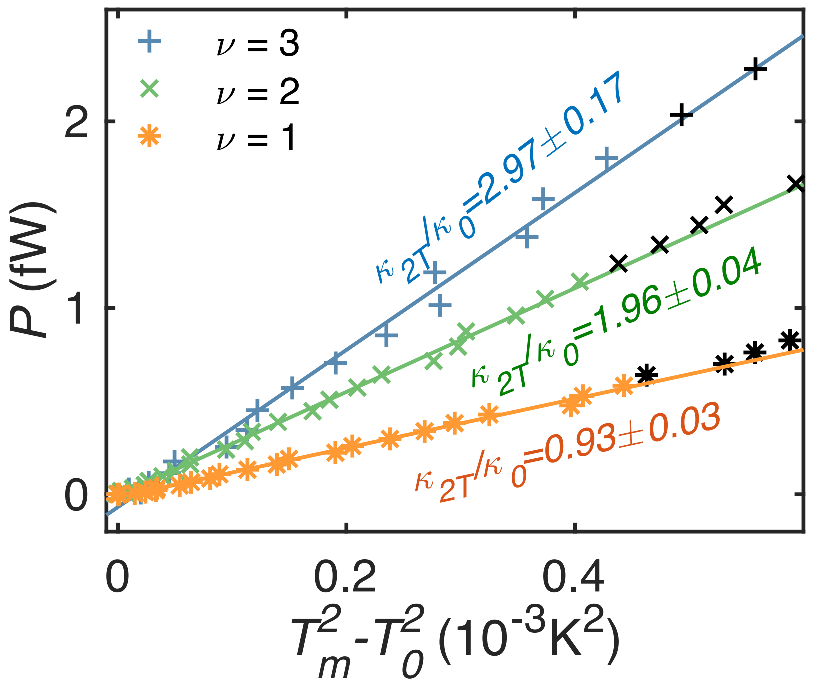

Supplementary Figure 3. **Extraction of the thermal conductance for integer states.** Injected power P vs $T_m^2 - T_0^2$, where T_m and T_0 are the Ohmic contact and base temperatures respectively. The colored markers (low temperature data $T_m < 25$mK) were linearly fitted to extract κ_{2T}. The black markers are for high temperature points, which were not included in the fit. Data is plotted for $\nu = 1, 2, 3$ in orange, green, and blue respectively. The slopes of the linear lines fit well to expected values of $\kappa_{2T}/\kappa_0 = n_d = 1, 2, 3$ respectively.

Supplementary Note 4. ESTIMATES OF THE THERMAL EQUILIBRATION AND DISSIPATION LENGTHS

In the main text, we demonstrated that the decay of the upstream noise with distance L is essentially the same for all considered filling fractions, and is well described by a simple exponential with the decay length $\approx 200\,\mu$m (see Fig. 2b in the main text). This observation strongly suggests that the dominant mechanism determining the decay of the upstream noise is dissipation (leakage) of the energy from the quantum-Hall edge to the environment, with the characteristic decay length $l_{\text{dis}} \approx 200\,\mu$m. At the same time, inter-mode thermal equilibration is not operative on the studied distances. In this section, we present further details of the analysis of the data and the fitting procedure supporting these conclusions.

In Supplementary Ref. 5, the authors derived, using a phenomenological model, a generic

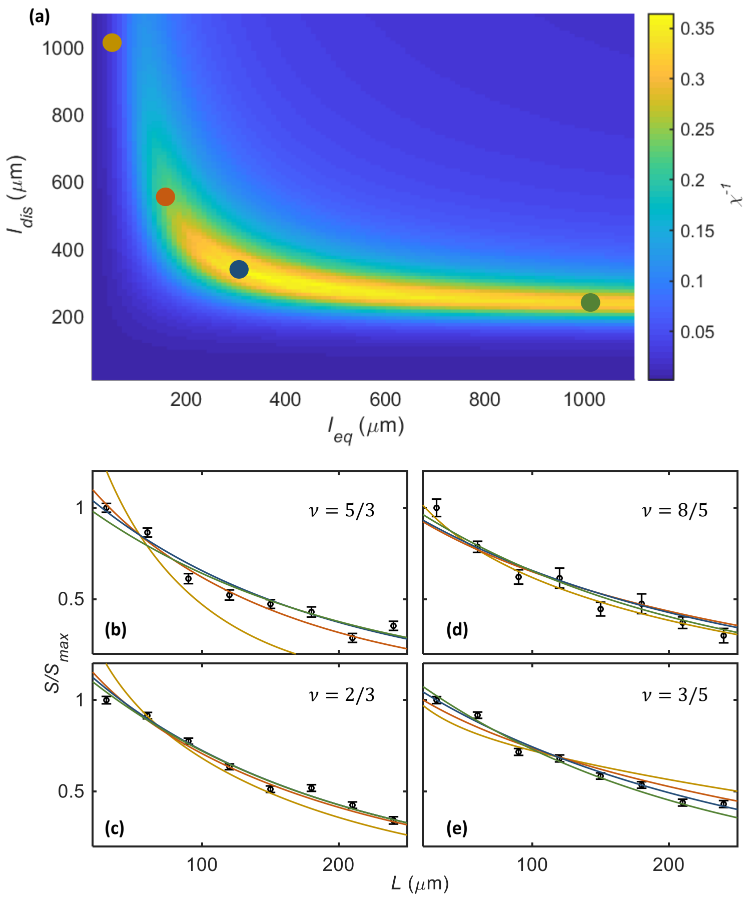

Supplementary Figure 4. **Estimation of thermal equilibration and dissipation lengths.** (a) Goodness of fits for the measured noise profiles, as a function of the equilibration length l_{eq} and the dissipation length l_{dis}. No good fit exists for $l_{\text{eq}} < 200\mu m$. On the other hand, our data is consistent with $l_{\text{eq}} \to \infty$ and $l_{\text{dis}} \approx 200\mu m$. The four colored points are at: $l_{\text{eq}} = 1050\mu m$, $l_{\text{dis}} = 220\mu m$ (green); $l_{\text{eq}} = 330\mu m$, $l_{\text{dis}} = 330\mu m$ (blue); $l_{\text{eq}} = 150\mu m$, $l_{\text{dis}} = 550\mu m$ (orange); $l_{\text{eq}} = 50\mu m$, $l_{\text{dis}} = 1050\mu m$ (yellow), and correspond to the four fits presented in (b-e). While the green and blue curves fit well to the measured data (markers in black with error bars representing the statistical error) at all filling factors, the orange curve fits poorly at $\nu = 3/5$, and the yellow curve fits poorly at $\nu = 5/3$ and $\nu = 3/5$.

formula for the temperature of upstream modes, $T_U(L)$ in the presence of equilibration and dissipation. When the upstream modes are sourced at a temperature T_m and the distance

is L, then

$$T_U^2(L) = T_0^2 + \frac{1}{2}\frac{\Lambda(T_m^2 - T_0^2)}{(N/(2\tilde{n}) + l_{\rm eq}/l_{\rm dis})\sinh[\Lambda L/l_{\rm eq}] + (\Lambda/2)\cosh[\Lambda L/l_{\rm eq}]} e^{-\frac{L}{\tilde{n}l_{\rm eq}}}. \quad (1)$$

Here, $\tilde{n} = (n_u n_d)/(n_d - n_u)$, $N = (n_u + n_d)/(n_d - n_u)$, and $\Lambda = \sqrt{\frac{1}{\tilde{n}^2} + 4\frac{l_{\rm eq}}{l_{\rm dis}}\left(\frac{N}{\tilde{n}} + \frac{l_{\rm eq}}{l_{\rm dis}}\right)}$. In order to have a quantitative bound on $l_{\rm eq}$, we fit our measured results from device A (Fig. 2b in the main text) to Supplementary Eq. (1). We assume that both $l_{\rm eq}$ and $l_{\rm dis}$ do not change between different states and that these parameters are the approximately the same for different modes on the same edge. We calculate the goodness of the fit of the model described by Supplementary Eq. (1) with different equilibration and dissipation lengths. The only parameter that we fit separately for each state is the noise amplitude at the shortest length (which is affected by microscopic properties of the hot-spot, and thereby beyond experimental control). The goodness of the fit is defined as

$$\chi^2(l_{\rm eq}, l_{\rm dis}) = \frac{1}{N}\sum \frac{(S(l_{\rm eq}, l_{\rm dis}, L) - S^U_{\rm excess}(L))^2}{\sigma(L)^2}, \quad (2)$$

where $S(l_{\rm eq}, l_{\rm dis}, L) \propto T_U(L)$ is the expected noise according to Supplementary Eq. (1), $S^U_{\rm excess}(L)$ is the measured noise, $\sigma(L)$ is the uncertainty in the noise, and $N = 32$ is the total number of measured points (eight points for each of four different filling factors). The sum is over all the different lengths of all the different fillings. In Supplementary Figure 4(a) we show $\chi^{-1} = (\chi^2)^{-1/2}$ as a function of $l_{\rm eq}$ and $l_{\rm dis}$. We see that our measurements are consistent with a very long equilibration length, and a dissipation length of roughly $200\mu m$. The goodness of the fit becomes poor for $l_{\rm eq} < 200\mu m$, for any value of $l_{\rm dis}$. The corresponding fits at four distinct points are presented in Supplementary Figure 4(b-e) for all the measured states.

Supplementary Note 5. ESTIMATION OF THE CHARGE EQUILIBRATION LENGTH

It was predicted in Supplementary Ref. 6 that the two-terminal electric conductance exhibits a crossover from the non-equilibrated to equilibrated value (e.g., from 4/3 to 2/3 at $\nu = 2/3$) when the length is increased. Such a crossover was subsequently observed experimentally in an engineered $\nu = 2/3$ edge[7]. In contrast, almost all previous experiments on conventional edges exhibited equilibrated values of the electric two-terminal conductance (i.e., $G_{2T} = \nu e^2/h$), indicating very short charge equilibration lengths. A slight deviation

in the conductance value indicating incomplete charge equilibration has been reported at the non-engineered $\nu = 2/3$ edge for very short edge distances[8]. In order to study the equilibration of charge in our devices, we sourced an AC voltage at the resonance frequency of the upstream amplifier (i.e., not a DC current like in the main measurements) from S_1 and measured the resulting voltage in the upstream amplifier. Given a source voltage V_S, and assuming that $G_U \ll G_D$, we get using standard Landauer-Büttiker formalism

$$V_{\text{amp}} = V_S \frac{G_U}{3G_{2T}}. \tag{3}$$

Here, G_U and G_D are the conductances from Ω_m to the upstream (downstream) amplifier, with $G_U + G_D = G_{2T}$. The factor of 3 in Supplementary Eq. (3) comes from the three arms of the device. For full charge equilibration, the entire charge current flows downstream. Thus, the ratio G_U/G_{2T} serves as a quantitative measure of deviation from full charge equilibration.

Our results for G_U/G_{2T} are shown in Supplementary Figure 5. For $\nu = 1$ and $\nu = 3$, we find $G_U/G_{2T} = 0$ as expected: for integer filling factors, the edge hosts only downstream modes, i.e. there is only downstream charge transport. By contrast, for $\nu = 2/3$ and $\nu = 3/5$, and for short propagation lengths, we observe non-zero values G_U/G_{2T}, indicating incomplete charge equilibration. However, the observed values of G_U/G_{2T} are very small even for the shortest length of 15μm. Specifically, we found $G_U/G_{2T} = 7 \times 10^{-3}$ and 3×10^{-4} for $\nu = 2/3$ and $\nu = 3/5$, respectively. This implies that these edges are nearly fully electrically equilibrated already for this short length, i.e., the charge equilibration length is substantially shorter than 15μm.

In order to rule out the possibility that the upstream current is a result of bulk currents due to finite longitudinal conductivity, we repeated the measurement at a higher temperature. We observed that G_U decreases when the temperature is raised to 21mK. This behavior is consistent with charge equilibration, since the charge equilibration length is expected to decrease with increasing temperature. At the same time, upstream transport via bulk currents would show an opposite behavior, as the longitudinal conductivity is expected to increase with temperature. Thus, the observed temperature dependence confirms that the non-zero values of G_U are due to incomplete electric equilibration between the counterpropagating modes. To estimate the value of the corresponding equilibration length l_{eq}^C, we recall[6,9] that the conductance approaches exponentially its limiting (non-equilibrated) value for $L \gg l_{\text{eq}}^C$.

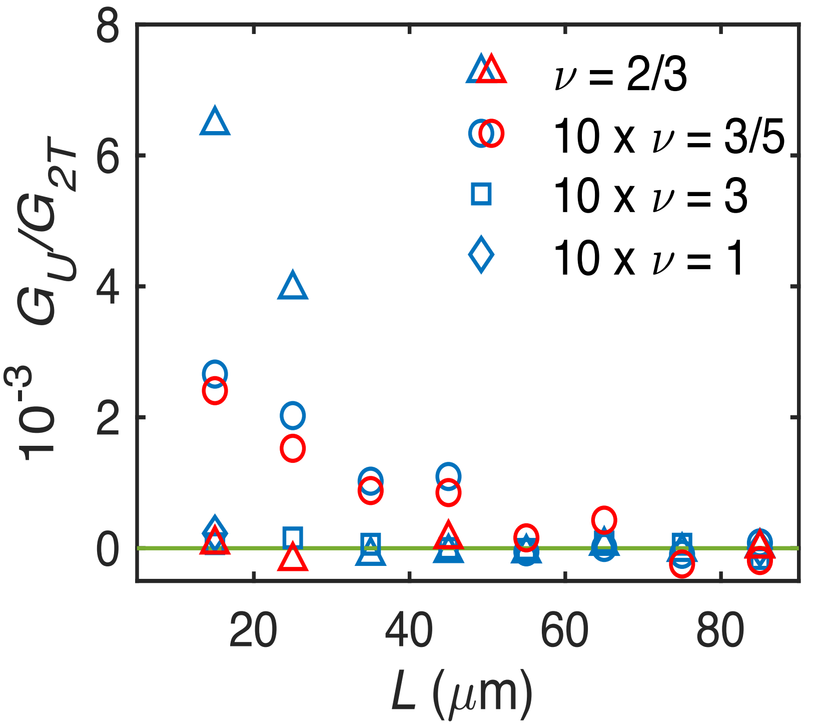

Supplementary Figure 5. **Estimation of charge equilibration lengths.** Upstream charge conductance G_U (normalized to $G_{2T} = \nu e^2/h$) vs propagation length L. When L is short, there is a finite upstream conductance for $\nu = 2/3$ and $\nu = 3/5$, but not for $\nu = 3$ and $\nu = 1$.The upstream conductance is always small (below $7 \times 10^{-3} G_{2T}$ for $\nu = 2/3$ and below $3 \times 10^{-4} G_{2T}$ for $\nu = 3/5$) and decays with L, in accordance with charge equilibration. At a higher temperature of $T_0 = 21$mK (red markers), G_U decreases (vanishes fully for $\nu = 2/3$), ruling out the possibility that the upstream current is a result of a finite longitudinal conductance. Blue markers denote a lower temperature $T = 11$mK for $\nu = 3$ and $\nu = 3/5$, $T = 13$mK for $\nu = 1$, $T = 14$mK for $\nu = 2/3$, respectively.

Thus, we estimate the L dependence of G_U for $\nu = 2/3$ and $\nu = 3/5$ via

$$G_U(L) = G_U(0)\, e^{-L/l_{\rm eq}^C}, \qquad (4)$$

where $G_U(0) = e^2\nu_-/h$ is the zero-length upstream conductance, and ν_- is the total filling factor of the upstream modes. We find $l_{\rm eq}^C \approx 4\mu$m for $\nu = 2/3$ (with $\nu_- = 1/3$) and $l_{\rm eq}^C \approx 2\mu$m for $\nu = 3/5$ ($\nu_- = 2/5$). These small values of the charge equilibration lengths stand in sharp contrast to the much larger estimate of the thermal equilibration length obtained in the main text.

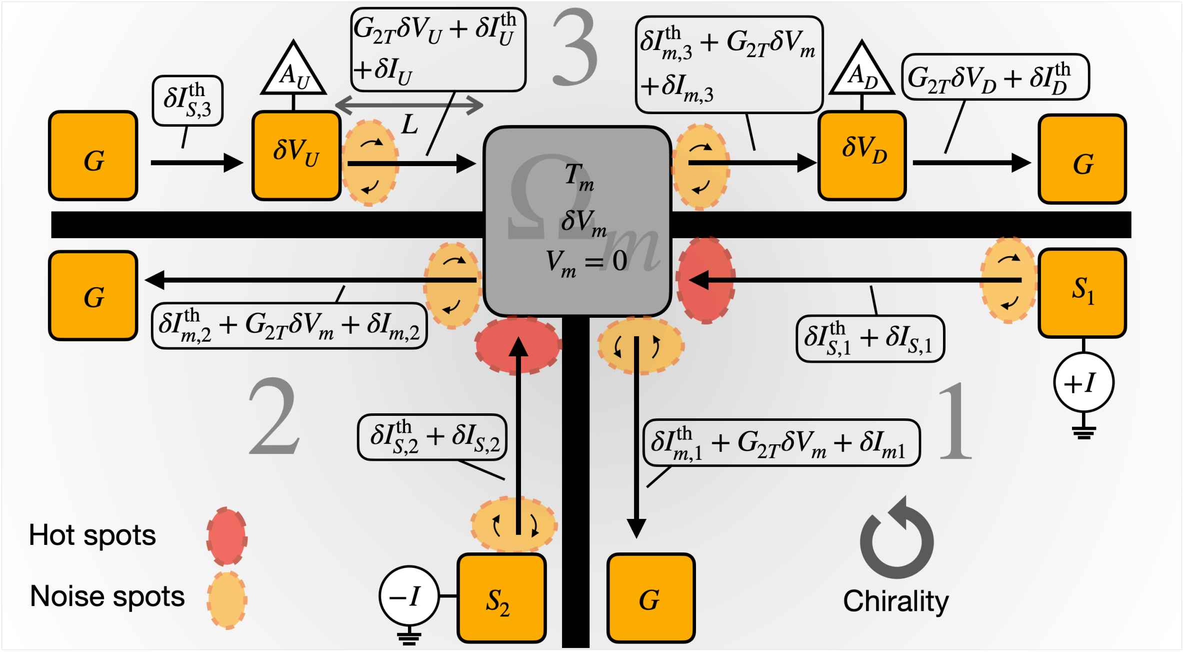

Supplementary Figure 6. **Device schematics**. The device consists of three arms (labels 1, 2, and 3), separated by insulating regions (black stripes) and connected to the central contact Ω_m. Black arrows denote the direction of charge flow along the edge states in each arm. Impinging currents $\pm I$ heat up Ω_m by Joule heating from voltage drops at the hot spots (red regions) and inside Ω_m. Partitioning of particle-hole pairs occurs at the noise spots (yellow regions). Excess noise is measured in contacts U (upstream from Ω_m) and D (downstream from Ω_m), with the help of amplifiers A_U and A_D. The upstream propagation length, between Ω_m and A_U, is denoted L. Current and voltage fluctuations are labelled δV and δI respectively. For the meaning of specific fluctuations, see Supplementary Note 6 B 1- Supplementary Note 6 B 2.

Supplementary Note 6. THEORETICAL MODEL OF THE DEVICE

A. Setup

In this Section, we theoretically model an experimental device of type $B1$ and $B2$ in the main text, as depicted in Supplementary Figure 6. The device consists of three arms (labelled $1-3$), separated by insulating regions and connected to a central floating contact Ω_m. Each arm is tuned to filling factor ν and the associated edge states have one incoming and one outgoing branch with respect to Ω_m and to the charge-flow direction (indicated by arrows in Supplementary Figure 6). We focus on such FQH states that exhibit counterpropagating

edge modes, so that each branch hosts both downstream and upstream modes. Assuming efficient charge equilibration, non-equilibrium charge currents flow only downstream (in the direction of arrows), which is in our convention the counter-clockwise direction in each of the arms (see the chirality sign in the bottom right corner of the figure). By contrast, we assume that the thermal equilibration between edge channels is very weak. On arm 3, there are two electrodes U and D, with attached amplifiers $\mathrm{A_U}$ and $\mathrm{A_D}$ (where subscripts indicate the upstream and downstream locations with respect to Ω_m), in which voltage/current fluctuations are measured. Except for Ω_m, all electrodes are assumed to be at the base temperature T_0.

The device is operated by the currents I_1 and I_2 biasing the arms 1 and 2 in source contacts $\mathrm{S_1}$ and $\mathrm{S_2}$, respectively. The source contact in the arm 3 is grounded, as marked by the label G in the figure. The two injected currents have equal magnitude but opposite sign: $I_1 = -I_2 \equiv I$. The average voltage V_m of the central contact and the injected electrical power P are then given as

$$V_m = \frac{I_1 + I_2}{2G_{2T}} = 0, \tag{5}$$

$$P = 2 \times \frac{I^2}{2G_{2T}} = \frac{I^2}{G_{2T}}, \tag{6}$$

where $G_{2T} = \nu e^2/h \equiv \nu G_0$. While $V_m = 0$, the floating electrode Ω_m is subject to voltage fluctuations δV_m.

In Supplementary Figure 6, we have also marked hot spots and noise spots. Hot spots are regions where the injected power dissipates and heat is generated. There are two such hot spots that heat Ω_m; in addition, a part of the heat is dissipated inside Ω_m. The noise spots are regions close to contacts where partitioning of charge due to edge impurity scattering may give rise to excess dc noise[10,11]. This happens if heat from hot spots or heated contacts reaches the noise spot. Due to the chiral nature of the edge and efficient charge equilibration, partitioning by scattering in regions other than noise spots does not contribute to the excess dc noise.

Details of the theoretical analysis of heat transport and noise in this setup are presented in the subsequent Sections. More specifically:

a) In Supplementary Note 7 we develop a microscopic model for the computation of noise, under the assumption of vanishing thermal equilibration between edge channels, and

derive formulas for the noise on an edge segment connecting two contacts with different temperatures.

b) In Supplementary Note 8 we use the results of Supplementary Note 7 to compute the downstream noise, i.e., the excess noise $S^{\mathrm{D}}_{\mathrm{excess}}$ in the downstream contact D (with amplifier $\mathrm{A_D}$). This result allows us extract the central-contact temperature T_m from experimental measurements of the noise.

c) In Supplementary Note 9 we use the results of Supplementary Note 7 to compute the upstream noise, i.e., the excess noise $S^{\mathrm{U}}_{\mathrm{excess}}$ in the upstream electrode U (with amplifier $\mathrm{A_U}$) as a function of T_m.

d) In Supplementary Note 10 we establish a power-balance relation between the injected electrical power P and the outgoing edge heat current $J^Q_{\mathrm{edge}} = 3\kappa_{2T}(T_m^2 - T_0^2)/2$. This relation, in combination with the results for the noise, allows us to extract experimentally the heat conductance $G^Q_{2T} = \kappa_{2T} T$ of the device (which is naturally measured in units of $\kappa_0 = \pi^2 k_B^2/3h$).

e) In Supplementary Note 11 we discuss theoretical predictions for the heat conductance κ_{2T}/κ_0 in the regime of vanishing heat equlibration.

f) A comparison of results from the theoretical analysis with the experiment is presented in Supplementary Note 12.

B. General analysis of downstream and upstream noise

As a starting point for the theory, we derive here expressions for the noise in the two amplifiers from a general analysis of the device.

1. Downstream noise

We begin by computing the downstream noise. We note first that, since we are interested in the noise at a low frequency ω_m, the condition of charge conservation at the central Ohmic contact Ω_m should be imposed. The charge conservation holds under the condition $(h/e^2)\omega_m C \ll 1$, where C is the capacitance of the island. Equivalently, this condition can

be written as $\omega_m \ll E_C$, where $E_C \sim e^2/C$ is the charging energy of Ω_m. Let us emphasize that, at the same time, the capacitance C is assumed to be sufficiently large such that $E_C \ll k_B T_0$. This ensures that Ω_m efficiently equilibrates impinging edge channels, so that heat Coulomb blockade[3,4] is not operative.

The incoming and outgoing current fluctuations on Ω_m are (see Supplementary Figure 6)

$$\delta I_m^{\rm in} = \left(\delta I_{S_1}^{\rm th} + \delta I_{S_1}\right) + \left(\delta I_{S_2}^{\rm th} + \delta I_{S_2}\right) + \delta I_U^{\rm th} + \delta I_U - \delta I_{m,1} - \delta I_{m,2} - \delta I_{m,3}, \tag{7}$$

$$\delta I_m^{\rm out} = 3G_{2T}\delta V_m + \delta I_{m,1}^{\rm th} + \delta I_{m,2}^{\rm th} + \delta I_{m,3}^{\rm th}. \tag{8}$$

In Supplementary Eqs. (7) and (8), δI_{S_i} are non-equilibrium current fluctuations impinging on Ω_m. These contributions originate from heat back-propagating from Ω_m to the noise spots (yellow regions) close to the source contacts[10,11]. Likewise, $\delta I_{m,i}$ and δI_U come from charge partitioning in the noise spots close to the central contact Ω_m and the upstream contact U. Finally, $\delta I_{S_i}^{\rm th}$, $\delta I_{m,i}^{\rm th}$, and $\delta I_U^{\rm th}$ are equilibrium current fluctuations from sources, the central contact Ω_m, and the upstream contact U, respectively. Their noise correlations are given by Johnson-Nyquist noise with their respective temperature.

As discussed in Supplementary Note 1, the upstream contact is assumed to be grounded at the frequency range where downstream noise is measured. Then, the current fluctuations from the upstream contact U read $\delta I_U^{\rm th} + \delta I_U$, which appear in Supplementary Eq. (7).

Equating (in view of charge conservation) $\delta I_m^{\rm in} = \delta I_m^{\rm out}$ and solving for $G_{2T}\delta V_m$, we find

$$G_{2T}\delta V_m = \frac{1}{3}\Big(\Delta I_{S_1} + \Delta I_{S_2} + \Delta I_U - \Delta I_{m,1} - \Delta I_{m,2} - \Delta I_{m,3}\Big), \tag{9}$$

where $\Delta I_i \equiv \delta I_i^{\rm th} + \delta I_i$ for $i = (S_1), (S_2), (m,1), (m,2), (m,3)$, or U. Next, by equating incoming and outgoing fluctuations at the downstream contact D, we find

$$\left(\delta I_{m,3}^{\rm th} + \delta I_{m,3}\right) + G_{2T}\delta V_m = G_{2T}\delta V_D + \delta I_D^{\rm th}, \tag{10}$$

where δV_D are the local voltage fluctuations and $I_D^{\rm th}$ are thermal fluctuations associated with the temperature T_0 of the contact D.

The excess downstream noise is defined as

$$S_{\rm excess}^{\rm D} = \overline{(G_{2T}\delta V_D)^2} - 4G_{2T}k_B T_0\,. \tag{11}$$

By inserting $G_{2T}\delta V_m$ from Supplementary Eq. (9) into Supplementary Eq. (10), solving for

$G_{2T}\delta V_D$, and substituting in Supplementary Eq. (11), we obtain

$$\begin{aligned} S^{\mathrm{D}}_{\mathrm{excess}} &= \frac{4}{9}\overline{(\Delta I_{m,3})^2} + \overline{\left(\delta I^{\mathrm{th}}_D\right)^2} + \frac{1}{9}\left[\overline{(\Delta I_{m,1})^2} + \overline{(\Delta I_{m,2})^2} + \overline{(\Delta I_{S_1})^2} + \overline{(\Delta I_{S_2})^2} + \overline{(\Delta I_U)^2}\right] \\ &\quad - 4G_{2T}k_BT_0 \\ &= \frac{2}{3}\overline{(\Delta I_m)^2} + \frac{1}{9}\left[S^{S_1}_{\mathrm{excess}} + S^{S_2}_{\mathrm{excess}} + S^{\mathrm{U}}_{\mathrm{excess}}\right] - \frac{4}{3}G_{2T}k_BT_0. \end{aligned} \tag{12}$$

Here we have used independence of different sources of fluctuations, which implies that the cross-correlations are zero. In the final equality, we assumed that $\overline{(\Delta I_{m,j})^2} \equiv \overline{(\Delta I_m)^2}$ are independent of j, i.e., equal for all three arms. We have also defined excess noises $S^i_{\mathrm{excess}} \equiv \overline{(\Delta I_i)^2} - 2G_{2T}k_BT_0$ for $i = (S_1)$, (S_2), and U. Furthermore, $\overline{\left(\delta I^{\mathrm{th}}_D\right)^2} = 2G_{2T}k_BT_0$.

Let us analyze the final form of Supplementary Eq. (12). The first two terms there represent two distinct contributions to the downstream noise. The first term is the thermal noise of Ω_m (see Supplementary Note 8 for a detailed discussion), while the second term results from the sum of excess noises from partitioning close to the upstream amplifier, $S^{\mathrm{U}}_{\mathrm{excess}}$ (see Supplementary Note 6 B 2 and Supplementary Note 9 for detailed discussions) and the source contacts, $S^{S_j}_{\mathrm{excess}}$. The last term (with the minus sign) is the subtraction of thermal noise (with temperature T_0), in correspondence with the definition of the excess noise.

If edge channels are thermally equilibrated (e.g., by impurity scattering), the Johnson-Nyquist relation holds for $\overline{(\Delta I_m)^2}$, yielding $\overline{(\Delta I_m)^2} = 2G_{2T}k_BT_m$. In this case we arrive at

$$S^{\mathrm{D}}_{\mathrm{excess}} = \frac{4}{3}k_BG_{2T}(T_m - T_0) + \frac{1}{9}\left[S^{S_1}_{\mathrm{excess}} + S^{S_2}_{\mathrm{excess}} + S^{\mathrm{U}}_{\mathrm{excess}}\right]. \tag{13}$$

Supplementary Eq. (13) establishes a relation between the downstream noise and $\Delta T \equiv T_m - T_0$. Therefore, it allows one to determine the temperature of the central contact Ω_m by measuring the downstream noise S^{D}_{excess}. The last term in Supplementary Eq. (13) is a relatively small correction (due to the factor 1/9). Still, it is appreciable and should be taken into account if one wants to find T_m with a good accuracy. This is done in the present work, when the formula generalizing Supplementary Eq. (13) on a non-equilibrated regime is used for determining T_m. The noise $S^{\mathrm{U}}_{\mathrm{excess}}$ is directly measured experimentally. The noises $S^{S_1}_{\mathrm{excess}}$ and $S^{S_2}_{\mathrm{excess}}$ can be also be obtained from measurements of $S^{\mathrm{U}}_{\mathrm{excess}}$, since the generation of upstream noise is fully analogous in all three arms.

In Supplementary Note 8, we microscopically compute $\overline{(\Delta I_m)^2}$ in Supplementary Eq. (12) for the case of the absence of thermal equilibration at the edge. We find how the Johnson-Nyquist formula for $\overline{(\Delta I_m)^2}$ should be corrected when the edge is not thermally equilibrated. This allows us to extend on such non-equilibrated regime the procedure of determining the temperature T_m by means of measuring the downstream noise $S^{\rm D}_{\rm excess}$.

2. *Upstream noise*

We now consider the upstream noise on arm 3, as measured in the contact U by means of the amplifier $A_{\rm U}$. Conservation of current fluctuations at the upstream contact U gives

$$-\delta I^{\rm U} + \delta I^{\rm th}_{S_3} = G_{2T}\delta V_U + \delta I^{\rm th}_U, \tag{14}$$

where $\delta I^{\rm th}_{S_3}$ are equilibrium fluctuations impinging from the top left grounded contact. Here we have taken into account that, under the used experimental design, the upstream contact U is floating at the frequency range where upstream noise is measured. Re-arranging, we get $G_{2T}\delta V_U = \delta I^{\rm th}_{S_3} - \delta I^{\rm th}_U - \delta I^{\rm U} = \delta I^{\rm th}_{S_3} - \Delta I_U$, so that the upstream excess noise is given by

$$\begin{aligned} S^{\rm U}_{\rm excess} &\equiv \overline{(G_{2T}\delta V_U)^2} - 4k_B G_{2T} T_0 = \overline{(\Delta I_U)^2} + \overline{(\delta I^{\rm th}_{S_3})^2} - 4G_{2T}k_B T_0 \\ &= \overline{(\Delta I_U)^2} - 2G_{2T}k_B T_0. \end{aligned} \tag{15}$$

Here, we used that all cross-correlations are zero and $\overline{\left(\delta I^{\rm th}_{S_3}\right)^2} = 2G_{2T}k_B T_0$. In Supplementary Eq. (15), $\overline{(\Delta I_U)^2}$ is the non-equilibrium noise generated by partitioning at the noise spot just to the right of the contact U. Equation (15) states that the upstream amplifier detects noise which is generated when heat flows from Ω_m and heats the noise spot. Hence, the upstream noise can be used as a local thermometer of the noise spot. Under the assumption that the contact U is acting as a heat reservoir (i.e., its temperature is kept fixed at T_0), the upstream excess noise is independent of other non-equilibrium noise sources in the device. In Supplementary Note 9, we compute $\overline{(\Delta I_U)^2}$ microscopically.

Supplementary Note 7. MICROSCOPIC MODEL AND CALCULATION OF NOISE ON AN EDGE SEGMENT

In the preceding Section, we derived general formulas for the excess noise. The key quantities there are $\overline{(\Delta I_m)^2}$ in Supplementary Eq. (12) for the downstream noise and $\overline{(\Delta I_U)^2}$

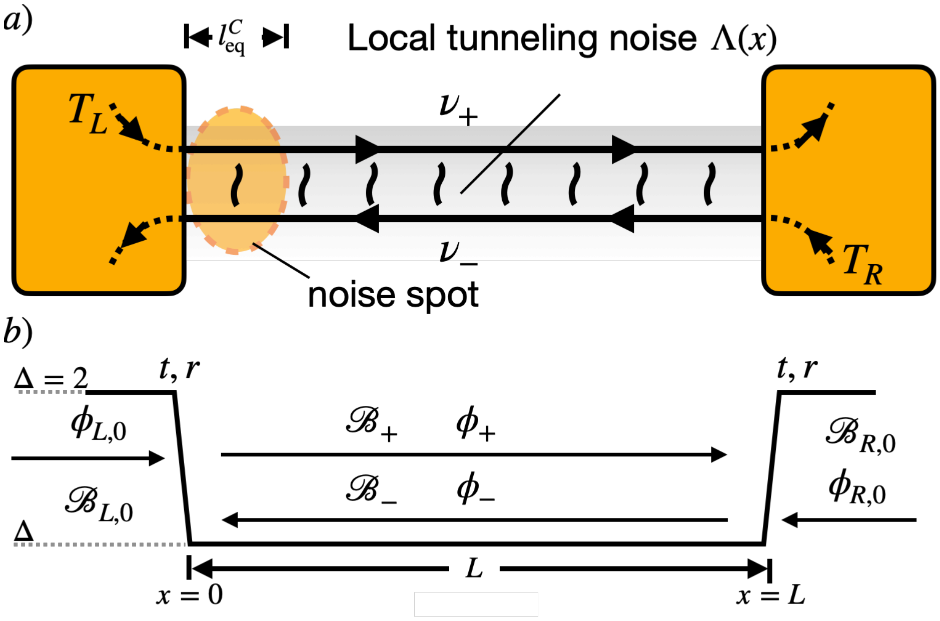

Supplementary Figure 7. **Noise generation on the edge.** a) The two contacts are at different temperatures T_L and T_R but at the same voltage. Excess dc noise is predominantly generated in the region of the spatial extent $\sim l_{\rm eq}^C$ (noise spot) close to the left contact, see Supplementary Eq. (16). b) Bosonic scattering states $\phi_{L,0}$ and $\phi_{R,0}$ emanating from the left and right contacts are characterized by equilibrium distribution functions $\mathcal{B}_{L,0}$ and $\mathcal{B}_{R,0}$ controlled by temperatures of the contacts. The distribution functions $\mathcal{B}_\pm$ of the eigenmode states $\phi_\pm$ in the interacting region are determined by $\mathcal{B}_{L,0}$ and $\mathcal{B}_{R,0}$ in combination with reflection (r) and transmission (t) amplitudes of bosons at boundaries between the contacts and the interacting region, see Supplementary Eqs. (30)-(31).

in Supplementary Eq. (15) for the upstream noise. Both these quantities represent a noise generated on an edge segment connecting two contacts with different temperatures: T_0 and T_m, with $T_m > T_0$. The only difference is that in the case of $\overline{(\Delta I_m)^2}$ the upstream contact Ω_m is hot and the downstream contact D is cold, while in the case of $\overline{(\Delta I_U)^2}$ the situation is opposite: the upstream contact U is cold, while the downstream contact Ω_m is hot. We are now going to compute the noise generated on a segment between the contacts with two different temperatures, T_L and T_R. For $T_L > T_R$ this will give $\overline{(\Delta I_m)^2}$, and for $T_L < T_R$ we will find $\overline{(\Delta I_U)^2}$.

To this end, we present in this Note a microscopic model to compute noise generated on a FQH edge segment connected to two contacts at $x = 0$ and $x = L$ (see Supplementary Figure

7a). To do so, we use a formalism developed in Supplementary Refs. 10–13. In accordance with experimental observations, we always assume full charge equilibration, $l_{\rm eq}^C \ll L$, where $l_{\rm eq}^C$ is the characteristic length scale (charge equlibration length) of inter-edge channel electron tunneling. However, in contrast to previous work[14], we are here interested in noise in the regime of no thermal equilibration between the edge channels. Weak thermal equlibration can be theoretically achieved with sufficiently strong inter-channel interactions[15]. We first focus on the $\nu = 2/3$ state, which is the archetypical hole-conjugate state, and whose edge hosts channels of both chiralities. Our approach is then generalized to other hole-conjugate states.

A. Filling 2/3

The edge segment consists of two counter-propagating edge channels, one downstream (+) associated with the corresponding filling factor discontinuity ν_+ and one upstream channel (-) associated with ν_-. In contrast to the standard Luttinger liquid ($\nu_+ = \nu_- = 1$), the chiral nature of the edge is captured by $\nu_+ > \nu_-$; downstream transport is taken from left to right. Specifically, $\nu_+ = 1$ and $\nu_- = 1/3$ for the $\nu = 2/3$ edge[16]. We divide the the edge segment into three regions: the left contact region, a central region, and the right contact region (see Supplementary Figure 7b). While the inter-channel interaction is assumed to be screened to zero in the contacts, it is finite in the central region (see Supplementary Ref. 17 for a detailed discussion on this assumption). The scaling dimension Δ of the inter-channel tunneling operator (to be discussed below) quantifies the strength of the inter-channel interaction; $\Delta = 2$ corresponds to non-interacting channels while $\Delta = 1$ corresponds to the regime of strongly interacting channels[16]. Supplementary Figure 7b shows $\Delta(x)$ as a function of position x due to the attached contacts. We assume that Δ varies sharply at the boundaries between the regions, which is justified at low frequencies[6]. The two contacts are taken at different temperatures T_L and T_R, but at the same electrochemical potential.

The zero frequency noise S in either of the two contacts is generally given as[11]

$$S = \frac{2e^2}{hl_{\rm eq}^C}\frac{\nu_-}{\nu_+}(\nu_+ - \nu_-)\int_0^L dx\, \Lambda(x) e^{-\frac{2x}{l_{\rm eq}^C}} + \frac{2e^2}{h}k_B T_L \frac{(\nu_+ - \nu_-)^2}{\nu_+}. \quad (16)$$

Here, the first term describes the noise resulting from inter-channel electron tunneling along the edge. The exponential factor in the integral is a result of the chiral charge transport,

and implies that the dominant noise contribution comes from a region of size $\sim l_{\rm eq}^C$ close to the left contact. We call this region the noise spot. The local noise generated by impurity scattering is described by the noise kernel $\Lambda(x)$ given by

$$\Lambda(x) \equiv \frac{S_{\rm loc}(x, T_L, T_R)}{2g_{\rm loc}(x, T_L, T_R)}, \tag{17}$$

where $S_{\rm loc}(x, T_L, T_R)$ and $g_{\rm loc}(x, T_L, T_R)$ is the local electron tunneling dc noise and the tunneling conductance, respectively. Most generically, $S_{\rm loc}$ and $g_{\rm loc}$ depend on microscopic details such as the inter-channel interaction, the edge disorder strength, the local voltage difference between the channels, and the edge channel energy distributions.

The second term in Supplementary Eq. (16) describes downstream propagating thermal fluctuations injected by the left contact. The thermal fluctuations injected by the right contact reaching the noise spot are suppressed by a factor that is exponential in $L/l_{\rm eq}^C \gg 1$ and thus can be safely neglected[10].

In the present work, we are interested in the case of no voltage bias and no thermal equilibration. In this situation, Supplementary Eq. (16) can be simplified. Specifically, Λ becomes independent of x, and the integration can be trivially performed. Further, Λ depends only on the temperatures T_L and T_R and the interaction parameter Δ. To emphasize this, we use the notation $\Lambda(T_L, T_R, \Delta)$ for the noise kernel. Thus, Supplementary Eq. (16) reduces to

$$S = \frac{e^2}{h}\frac{\nu_-}{\nu_+}(\nu_+ - \nu_-)\Lambda(T_L, T_R, \Delta) + \frac{2e^2}{h}k_B T_L \frac{(\nu_+ - \nu_-)^2}{\nu_+}. \tag{18}$$

Our goal is now to use Supplementary Eqs. (17) and (18) to compute the excess charge noise generated by the temperature difference, $T_L \neq T_R$, in the absence of a net charge current flow. This type of noise is called "ΔT noise", and has in recent years attracted increasing attention, both from theoretical[18–24] and experimental[25–28] points of view.

Supplementary Eqs. (17) and (18) are applicable to both downstream and upstream noise (see Supplementary Figure 6). As discussed above, the upstream noise corresponds to $T_L = T_0$ and $T_R = T_m$, i.e., to the right contact being hot, $T_R > T_L$ (the edge segment on arm 3 to the left of Ω_m in Supplementary Figure 6). Conversely, the downstream noise corresponds to $T_L = T_m$ and $T_R = T_0$, i.e., to a hot left contact, $T_L > T_R$ (the edge segment on arm 3 to the right of Ω_m in Supplementary Figure 6).

The expectation values of the local noise and electron tunneling conductance can be computed within the chiral Luttinger liquid model[29]. To leading order in the tunneling

strength Γ_0 (that is assumed for simplicity uniform), these quantities are given as[15,21]

$$S_{\rm loc} = 4\int_{-\infty}^{\infty} d\tau \langle \mathcal{T}(\tau,0)\mathcal{T}^\dagger(0,0)\rangle, \tag{19}$$

$$g_{\rm loc} = 2i\int_{-\infty}^{\infty} d\tau\tau \langle \mathcal{T}(\tau,0)\mathcal{T}^\dagger(0,0)\rangle. \tag{20}$$

Here

$$\mathcal{T}(\tau,0) = \frac{\Gamma_0}{2\pi b}\exp\left[i\sqrt{\Delta-1}\phi_+(\tau,0) + i\sqrt{\Delta+1}\phi_-(\tau,0)\right] \tag{21}$$

is the local tunneling operator expressed in terms of the bosonic eigenmodes $\phi_\pm$ in the interacting region[15], and b is a short distance cutoff. Evaluating the correlation function, we find

$$\langle \mathcal{T}(\tau,0)\mathcal{T}^\dagger(0,0)\rangle = \frac{|\Gamma_0|^2}{(2\pi b)^2} G_+(\tau,0)^{\Delta-1} G_-(\tau,0)^{\Delta+1}, \tag{22}$$

where

$$G_\pm(\tau,0) = \exp\left[\langle\phi_\pm(\tau,0)\phi_\pm(0,0)\rangle - \langle\phi_\pm(0,0)\phi_\pm(0,0)\rangle\right] \equiv \exp\left[\mathcal{B}_\pm(\tau,0)\right]. \tag{23}$$

The Fourier transform of $\mathcal{B}_\pm(\tau,0)$ in frequency space, $\mathcal{B}_\pm(\omega,0)$, corresponds to the distribution functions of the eigenmodes $\phi_\pm$. In the absence of thermal equilibration, these are non-equilibrium distribution functions, arising from scattering of bosonic modes at the boundaries between contacts and the interacting middle region (see Supplementary Figure 7b). We therefore now seek to express $\mathcal{B}_\pm$ in terms of the known equilibrium Bose distributions $\mathcal{B}_{L/R,0}$ of the non-interacting bosons $\phi_{L/R,0}$ in the contacts. Our approach follows closely that in Refs. 6 and 30. The bosonic states in the central region $\phi_\pm(\tau,x)$ can be expanded as

$$\phi_+(\tau,x) = t\sum_n r^{2n}\phi_{L,0}(\tau-2n\tilde{\tau},x) + tr\sum_n r^{2n}\phi_{R,0}(\tau-2n\tilde{\tau},x),$$
$$\phi_-(\tau,x) = t\sum_n r^{2n}\phi_{R,0}(\tau-2n\tilde{\tau},x) + tr\sum_n r^{2n}\phi_{L,0}(\tau-2n\tilde{\tau},x). \tag{24}$$

Here, the parameter $\tilde{\tau} = \frac{L}{2}(v_+^{-1} + v_-^{-1})$ is the mean "flight-time" through the interacting region[6] and v_+ and v_- are the velocities of $\phi_\pm$. The parameters t and r are transmission and reflection coefficients due to the sharp change in interaction strength at the boundaries. The coefficients are given as

$$t = \frac{2(1-c^2)^{1/4}}{\sqrt{1-c}+\sqrt{1+c}}, \tag{25}$$

$$r = \frac{1-\sqrt{1-c^2}}{c}, \tag{26}$$

where c is related to Δ as

$$\Delta = \frac{2 - \sqrt{3}c}{\sqrt{1-c^2}}. \tag{27}$$

It follows from Supplementary Eq. (24) that

$$\mathcal{B}_\pm(\omega, 0) = t^2 \sum_{n,m}^{\infty} r^{2(n+m)} e^{2i(m-n)\omega\tilde{\tau}} \mathcal{B}_{L/R}(\omega, 0) + t^2 r^2 \sum_{n,m}^{\infty} r^{2(n+m)} e^{2i(m-n)\omega\tilde{\tau}} \mathcal{B}_{R/L}(\omega, 0), \tag{28}$$

where $\mathcal{B}_{R/L}(\omega, 0)$ is the Fourier transform of

$$\mathcal{B}_{R/L}(\tau, 0) = \langle \phi_{R/L}(\tau, 0)\phi_{R/L}(0, 0)\rangle - \langle \phi_{R/L}(0, 0)\phi_{R/L}(0, 0)\rangle. \tag{29}$$

We now neglect terms of the form $e^{2im\tilde{\tau}\omega}$ which arise due to the Fabry-Perot interference of bosonic modes reflected at boundary. These terms lead to an oscillatory behavior in energy on the scale $\pi v_\pm/L$. These oscillations will however be suppressed at temperatures $T_L, T_R \gg \pi v_\pm/L$, which is assumed as follows. In this case, Supplementary Eq. (28) reduces to

$$\mathcal{B}_+(\omega, 0) = \frac{T}{1-R^2}\mathcal{B}_L(\omega, 0) + \frac{TR}{1-R^2}\mathcal{B}_R(\omega, 0), \tag{30}$$

$$\mathcal{B}_-(\omega, 0) = \frac{T}{1-R^2}\mathcal{B}_R(\omega, 0) + \frac{TR}{1-R^2}\mathcal{B}_L(\omega, 0). \tag{31}$$

with $T = t^2$, $R = r^2$. Substituting the relations (30)-(31) into Supplementary Eq. (23), and the result into Supplementary Eq. (22), we express the tunneling correlation function as

$$\langle \mathcal{T}(\tau, 0)\mathcal{T}^\dagger(0, 0)\rangle = \frac{|\Gamma_0|^2}{(2\pi b)^2} G_L(\tau, 0)^{2d_L} G_R(\tau, 0)^{2d_R}, \tag{32}$$

where $G_L(\tau, 0)$ and $G_R(\tau, 0)$ are the equilibrium free-fermion Green's functions,

$$G_{L,R}(\tau, 0) = \frac{\pi b T_{L,R}/v_{L,R}}{\sin\left[\frac{\pi T_{L,R}}{v_{L,R}}(b - i\tau v_{L,R})\right]}. \tag{33}$$

Here, $v_{L,R}$ are the velocities of the bosonic modes in the contacts. At zero temperature, $G_{L,R}$ reduce to

$$G_{L,R}(\tau, 0) = \frac{b}{b - i\tau v_{L,R}}. \tag{34}$$

The exponents d_L and d_R in Supplementary Eq. (32) capture the interaction dependence of the tunneling. They are given in terms of Δ by the following expressions:

$$2d_L = \frac{\Delta^3 + \Delta - \sqrt{3(\Delta^2 - 1)}}{\Delta^2 + 3}, \tag{35}$$

$$2d_R = \frac{\Delta^3 + 5\Delta + \sqrt{3(\Delta^2 - 1)}}{\Delta^2 + 3}. \tag{36}$$

The Fourier transforms of the factors $G_{L,R}(\tau,0)^{2d_{L,R}}$ read

$$P_{L,R}(\omega,T_{L,R}) = \int_{-\infty}^{\infty} d\tau e^{i\omega\tau} G_{L,R}(\tau,0)^{2d_{L,R}}$$
$$= \left(\frac{2\pi b T_{L,R}}{v_{L,R}}\right)^{2d_{L,R}-1} \left(\frac{b}{v_{L,R}}\right) e^{\omega/(2T_{L,R})} \frac{|\Gamma(d_{L,R} + i\frac{\omega}{2\pi T_{L,R}})|^2}{\Gamma(2d_{L,R})}. \tag{37}$$

In the zero-temperature limit, $T_{L,R} \to 0$, they reduce to

$$P_{L,R}(\omega,0) = \int_{-\infty}^{\infty} d\tau e^{i\omega\tau} G_{L,R}(\tau,0)^{2d_{L,R}} = \frac{2\pi (b/v_{L,R})^{2d_{L,R}} \omega^{2d_{L,R}-1}\Theta(\omega)}{\Gamma(2d_{L,R})}. \tag{38}$$

Here, $\Gamma(z)$ is the gamma-function, and $\Theta(\omega)$ is the Heaviside step function.

With the Fourier transforms, the noise (19) can be expressed as

$$S_{\text{loc}} = \frac{4|\Gamma_0|^2}{(2\pi b)^2} \int_{-\infty}^{\infty} \frac{d\omega}{2\pi} P_L(-\omega,T_L) P_R(\omega,T_R), \tag{39}$$

and the tunneling conductance (20) as

$$g_{\text{loc}} = \frac{2|\Gamma_0|^2}{(2\pi b)^2} \frac{\partial}{\partial\omega'} \left(\int_{-\infty}^{\infty} \frac{d\omega}{2\pi} P_L(\omega'-\omega,T_L) P_R(\omega,T_R) \right) \Bigg|_{\omega'=0}. \tag{40}$$

Combining Supplementary Eqs. (39) and (40), we express the noise kernel (17) as

$$\Lambda(T_L,T_R,\Delta) = \frac{\int_{-\infty}^{\infty} d\omega P_L(-\omega,T_L) P_R(\omega,T_R)}{\frac{\partial}{\partial\omega'} \left(\int_{-\infty}^{\infty} d\omega P_L(\omega'-\omega,T_L) P_R(\omega,T_R) \right) \Big|_{\omega'=0}}. \tag{41}$$

Let us briefly recapitulate the procedure leading to Supplementary Eq. (41) for the noise kernel Λ. In this equation, the numerator and the denominator are, respectively, the local noise and the conductance of electron tunneling between two edge channels out of equilibrium. The bosonic scattering theory has allowed us to express these quantities in terms of known equilibrium distributions in the contacts. We recall that the situation of no thermal equilibration considered here is complementary to the limit of strong thermal equilibration, for which the local noise kernel can be expressed in terms of local edge channel temperatures[10,12]. Let us further emphasize that Λ depends on the edge interaction parameter Δ through the exponents $2d_{L,R}$ [see Supplementary Eqs. (35)-(36)] in the expressions for $P_{L,R}$ [see Supplementary Eq. (37)-(38)].

Substituting the expression (41) into the noise formula (18), we arrive at the final expression for the noise on the 2/3 edge:

$$S = \frac{2}{9}\frac{e^2}{h} \times \Lambda(T_L,T_R,\Delta) + \frac{8}{9}\frac{e^2}{h} k_B T_L. \tag{42}$$

It is instructive to inspect Supplementary Eq. (42) in the case of global equilibrium, $T_L = T_R = T_0$. In this situation, we can analytically perform the integrals in Supplementary Eq. (41) to obtain $\Lambda(T_0, T_0, \Delta) = 2k_B T_0$, independent of Δ. Then, Supplementary Eq. (42) reduces (as expected) to the Nyquist-Johnson relation

$$S = 2G_{2T} k_B T_0, \quad G_{2T} = \frac{2}{3}\frac{e^2}{h}. \tag{43}$$

Further, we briefly consider the regime of strong thermal equilibration. Then both edge channels equilibrate to T_L at the noise spot. In this case, the measured downstream noise is the Johnson-Nyquist noise at temperature T_L, i.e,

$$S = 2G_{2T} k_B T_L, \quad G_{2T} = \frac{2}{3}\frac{e^2}{h}. \tag{44}$$

B. Filling 3/5

The edge at filling $\nu = 3/5$ consists of three modes. One downstream mode ϕ_1 with filling factor discontinuity $\nu_1 = 1$ and two upstream modes $\phi_{1/3}$, $\phi_{1/15}$ with $\nu_{1/3} = 1/3$ and $\nu_{1/15} = 1/15$ respectively[31,32]. To greatly simplify the noise analysis of this edge, we now make the following assumptions. First, we assume that the equilibration lengths (for both charge and heat) of the two upstream modes is very small. We thus "merge" these two modes into one effective upstream mode. Secondly, we assume that both inter-mode tunneling and the inter-mode interaction is dominated by that between ϕ_1 and $\phi_{1/3}$. This assumption is based on that these modes are spatially closer than ϕ_1 and $\phi_{1/15}$. This can be motivated, e.g., within the composite fermion picture[33]. Roughly, in this picture, ϕ_1, $\phi_{1/3}$, and $\phi_{1/15}$ can be viewed as originating respectively from the zeroth, first, and second Landau levels of composite fermions. Hence, $\phi_{1/15}$ is situated closest to the bulk. Additionally, tunneling and interactions between the co-propagating $\phi_{1/3}$ and $\phi_{1/15}$ do not influence the equilibration or the heat conductance. Similar assumptions for the $\nu = 3/5$ edge were made recently in Supplementary Ref. 34, with excellent agreement between theory and experiment. With these simplifications, the noise kernel in Supplementary Eq. (16) becomes the same for $\nu = 2/3$ and $\nu = 3/5$ since they are based on the same single tunneling operator [see Supplementary Eq. (21)]. The difference in the generated noise for these fillings, in the thermally non-equilibrated regime, is the values of $\nu_\pm$. While $\nu = 2/3$, we have $\nu_+ = 1$ and $\nu_- = 1/3$, for $\nu = 3/5$, we have instead $\nu_+ = 1$ and $\nu_- = 1/3 + 1/15 = 2/5$. With these

parameters, we have obtain the noise for $\nu = 3/5$ as

$$S = \frac{6}{25}\frac{e^2}{h} \times \Lambda(T_L, T_R, \Delta) + \frac{18}{25}\frac{e^2}{h} k_B T_L. \tag{45}$$

For global equilibrium, we recover $S = 2G_{2T}k_BT_0$ with $G_{2T} = (3/5)e^2/h$, and for full thermal equilibration at temperature T_L in the noise spot, we find $S = 2G_{2T}k_BT_L$.

In the following two Sections, we use Supplementary Eqs. (42) and (45) to compute downstream and upstream noise in the case of no thermal equilibration.

Supplementary Note 8. CALCULATION OF DOWNSTREAM NOISE

In this Note, we use the results of Supplementary Note 7 to compute the downstream noise on an edge segment. The downstream noise configuration corresponds to choosing $T_L = T_m = T_0 + \Delta T$ and $T_R = T_0$ with $\Delta T > 0$.

We may then identify the noise in Supplementary Eq. (42) with the downstream noise $\overline{(\Delta I_m)^2}$ from Supplementary Eq. (12). We then have for $\nu = 2/3$

$$\overline{(\Delta I_m)^2} = \frac{2}{9}\frac{e^2}{h} \times \Lambda(\Delta T + T_0, T_0, \Delta) + \frac{8}{9}\frac{e^2}{h} k_B(\Delta T + T_0). \tag{46}$$

In Supplementary Figure 8a, we plot $\overline{(\Delta I_m)^2}$ as a function of ΔT for $T_0 = 14$ and various values of Δ. We choose this value of the base temperature T_0 for convenience of comparison to our experiment: T_0 was measured to be 14 mK for $\nu = 2/3$. We set the prefactor $(e^2/h)k_B$ in the expression for $\overline{(\Delta I_m)^2}$ to be unity. As a result, $\overline{(\Delta I_m)^2}$ is measured in the same units as the temperature.

We first note that the Δ-dependence of $\Lambda(T_0 + \Delta T, T_0, \Delta)$ is rather weak, within a few percent. It becomes especially weak for not too large ΔT, i.e., $\Delta T \lesssim 30$. In this regime, an excellent approximation to $\Lambda(T_0 + \Delta T, T_0, \Delta)$ is obtained by expanding Supplementary Eq. (42) to first order in ΔT for $\Delta = 1$. The result is the simple expression

$$\Lambda(T_0 + \Delta T, T_0, \Delta) \approx 2T_0 + 0.5\Delta T. \tag{47}$$

The corresponding result for the noise $\overline{(\Delta I_m)^2}$ is shown by the blue dashed line in Supplementary Figure 8a. In the same plot, we present (by black dashed line) the result for $\overline{(\Delta I_m)^2}$ obtained by using the large-ΔT asymptotics $\Lambda(\Delta T + T_0, 0, \Delta = 1) \simeq 0.948(\Delta T + T_0)$, which is a very good approximation for $\Delta T \gtrsim 20$. Further, the red line displays the Nyquist-Johnson noise $2G_{2T}k_B(\Delta T + T_0)$, which is obtained for $\overline{(\Delta I_m)^2}$ in the limit of full thermal

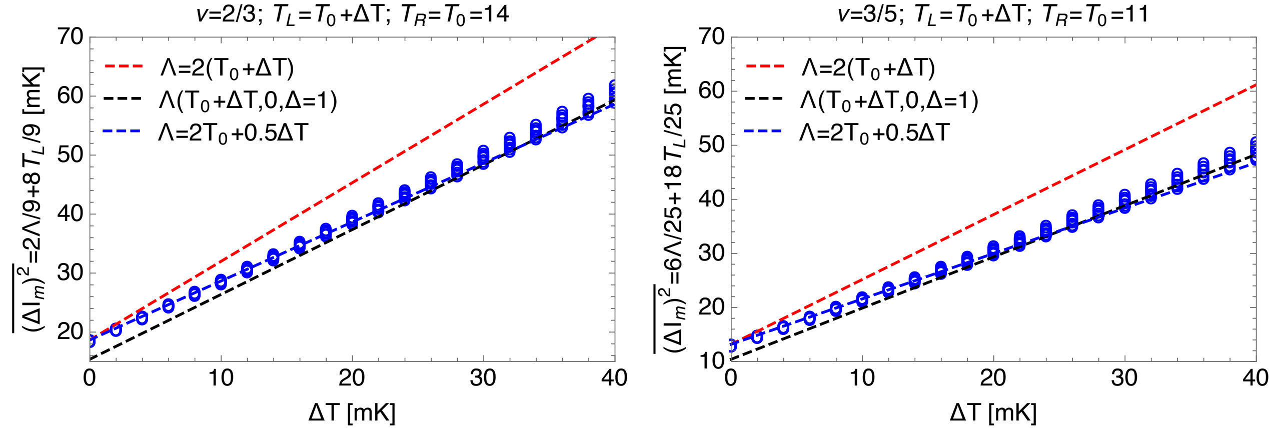

(a) (b)

Supplementary Figure 8. **Calculation of downstream noise.** Downstream noise, $\overline{(\Delta I_m)^2}$ [see Supplementary Eqs. (46) and (48)], as functions of $\Delta T = T_L - T_0$ in units of mK. We set $e^2/h = k_B = 1$ so that $\overline{(\Delta I_m)^2}$ is also measured in mK. (a) For $\nu = 2/3$, with $T_0 = 14$ corresponding to the experimentally measured value $T_0 = 14$ mK at this filling. The blue circles correspond to Δ varying from 1 (bottom) to 2 (top). The blue dashed line, Supplementary Eq. (47), is an excellent approximation for $0 \lesssim \Delta T \lesssim 30$. The black dashed line is obtained by using the large-ΔT asymptotics $\Lambda(\Delta T + T_0, 0, \Delta = 1) \simeq 0.948(\Delta T + T_0)$. The red dashed line is Johnson-Nyquist noise with the temperature $T_0 + \Delta T$ [see Supplementary Eq. (44)], for which $\overline{(\Delta I_m)^2} = 4(\Delta T + T_0)/3 = 2G_{2T}(T_0 + \Delta T)$ with $G_{2T} = 2/3$; this corresponds to the limit of full thermal equilibration. (b) Similar to (a) but at $\nu = 3/5$, with $T_0 = 11$ corresponding to the measured $T_0 = 11$ mK at this filling. The red, dashed line is Johnson-Nyquist noise $2G_{2T}(T_0 + \Delta T)$ with $G_{2T} = 3/5$.

equilibration, see Supplementary Eq. (44). Quite remarkably, the difference between the limits of no equilibration and full equilibration is rather small, within $\approx 20\%$. In the intermediate case of partial thermal equilibration, the results are expected to be in between. This weak dependence of the downstream noise on the degree of thermal equilibration, in combination with its very weak dependence on Δ in the non-equilibrated regime is highly favorable for using the downstream noise as a thermometer for measuring T_m. Indeed, even if the degree of thermal equilibration and the value of Δ are not known, one can use one of the limiting formulas (for the full equilibration or no equilibration) and obtain in any case a result for T_m with a reasonable accuracy.

Turning next to $\nu = 3/5$, we have from Supplementary Eq. (45) that the downstream

fluctuations become

$$\overline{(\Delta I_m)^2} = \frac{6}{25}\frac{e^2}{h} \times \Lambda(\Delta T + T_0, T_0, \Delta) + \frac{18}{25}\frac{e^2}{h} k_B(\Delta T + T_0). \tag{48}$$

This is plotted in Supplementary Figure 8b for $T_0 = 11$, since the base temperature at $\nu = 3/5$ was measured to 11 mK. The conclusions of the analysis is the same as those for $\nu = 2/3$.

Supplementary Note 9. CALCULATION OF UPSTREAM NOISE

In this Note, we use the results of Supplementary Note 7 to compute the upstream noise on an edge segment. The upstream noise configuration corresponds to the choice $T_R = T_m = T_0 + \Delta T$ and $T_L = T_0$ with $\Delta T > 0$. With this choice of parameters, we identify Supplementary Eq. (42) with the upstream noise $\overline{(\Delta I_U)^2}$ from Supplementary Eq. (15) and obtain for $\nu = 2/3$

$$\overline{(\Delta I_U)^2} = \frac{2}{9}\frac{e^2}{h} \times \Lambda(T_0, \Delta T + T_0, \Delta) + \frac{8}{9}\frac{e^2}{h} k_B T_0. \tag{49}$$

We plot $\overline{(\Delta I_U)^2}$ as a function of ΔT for various Δ in Supplementary Figure 9a. We note that similar to the downstream noise, the Δ-dependence is very weak, especially for small ΔT. In the interval $0 \lesssim \Delta T \lesssim 30$, the perturbative expansion in ΔT,

$$\Lambda(T_0, T_0 + \Delta T, \Delta) \approx 2T_0 + 1.5\Delta T, \tag{50}$$

which is shown by blue dashed line, serves as an excellent approximation for $\Lambda(T_0, T_0 + \Delta T, \Delta)$. For $\Delta T \gtrsim 30$, the large-ΔT asymptotics, $\Lambda(0, T_0 + \Delta T, \Delta = 1) \simeq 1.72(T_0 + \Delta T)$ (black dashed line) is a better approximation. Note that both asymptotics are actually rather close to each other and in fact serve as very good approximations in the whole range of ΔT. Comparing Supplementary Figure 8 and Supplementary Figure 9, we see that, for given ΔT, the upstream noise $\overline{(\Delta I_U)^2}$ is weaker than the downstream noise $\overline{(\Delta I_m)^2}$. The main reason is the second term in Supplementary Eq. (18) which corresponds to "hot fluctuations" for the downstream noise but to "cold fluctuations" for the upstream noise. This asymmetry between downstream and upstream "ΔT noise" is a consequence of the chiral nature of the edge.

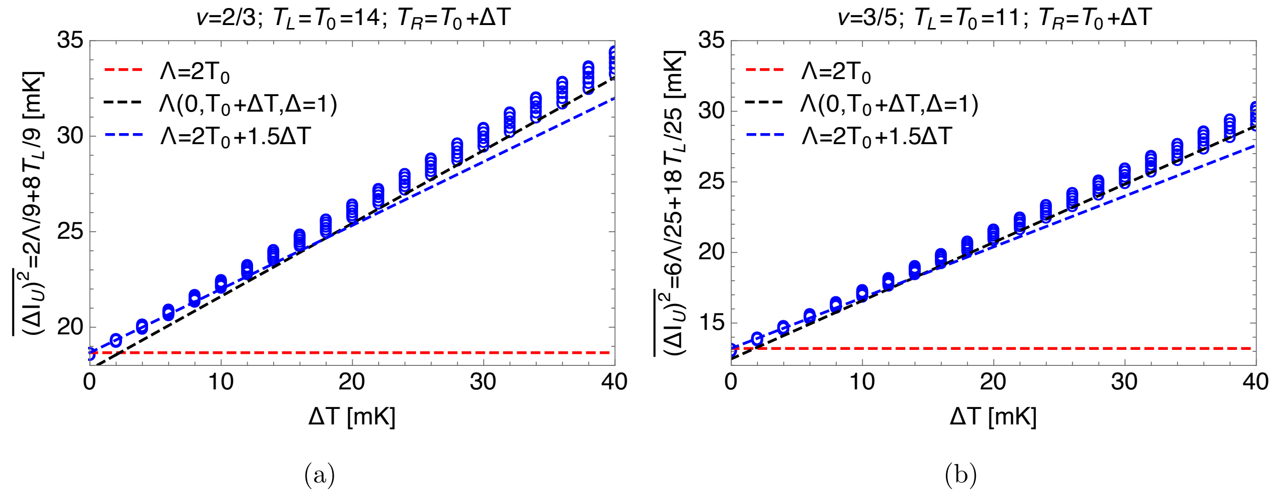

Supplementary Figure 9. **Calculation of upstream noise.** Upstream noise, $\overline{(\Delta I_U)^2}$, as a function of $\Delta T = T_R - T_0$ [see Supplementary Eqs. (49) and (51)] in units of mK. We set $e^2/h = k_B = 1$ so that $\overline{(\Delta I_U)^2}$ also is in units of mK. (a) For $\nu = 2/3$, with the base temperature $T_0 = 14$, corresponding to the measured $T_0 = 11$ mK at this filling. The blue circles correspond to Δ between 1 and 2. The blue dashed line, Supplementary Eq. (50), is an excellent approximation for $0 \lesssim \Delta T \lesssim 30$. The black dashed line is the large-ΔT asymptotics $\Lambda(0, \Delta T + T_0, \Delta = 1) \simeq 1.72(T_0 + \Delta T)$. The red dashed line is the Johnson-Nyquist noise with the temperature T_0 [see Supplementary Eq. (44)], for which $\overline{(\Delta I_m)^2} = 4T_0/3$; this corresponds to the limit of full thermal equilibration. (b) Similar to (a) but at $\nu = 3/5$, with $T_0 = 11$ corresponding to the measured $T_0 = 11$ mK at this filling. The red, dashed line is Johnson-Nyquist noise $\overline{(\Delta I_m)^2} = 2G_{2T}T_0$ with $G_{2T} = 3/5$.

A similar analysis for $\nu = 3/5$ gives, using Supplementary Eq. (18), the upstream noise

$$\overline{(\Delta I_U)^2} = \frac{6}{25}\frac{e^2}{h} \times \Lambda(T_0, \Delta T + T_0, \Delta) + \frac{18}{25}\frac{e^2}{h} k_B T_0. \tag{51}$$

This is plotted for $\nu = 3/5$ in Supplementary Figure 9b. Similarly to $\nu = 2/3$, the approximation (50) is excellent in the considered temperature regime.

Supplementary Note 10. POWER BALANCE EQUATION AND THEORETICAL FOUNDATIONS OF EXPERIMENTAL DETERMINATION OF HEAT CONDUCTANCE.

In this Note, we present theoretical foundations for experimental determination of the heat conductance κ_{2T} on the basis of measurements of the noises $S^{\mathrm{D}}_{\mathrm{excess}}$ and $S^{\mathrm{U}}_{\mathrm{excess}}$ as functions of the heating current I.

The heat conductance κ_{2T} of the device is obtained by the principle developed in Supplementary Ref. 35 and applied to FQH states in Supplementary Refs. 14, 15, and 36. The key principle at work is that, in the steady state, a power P_{diss} is dissipated in Ω_m, which in turn is evacuated by heat currents carried by phonons, J^Q_{phonons}, and edge states, J^Q_{edge}. This relation is captured by the power balance equation

$$P_{\mathrm{diss}} = J^Q_{\mathrm{edge}} + J^Q_{\mathrm{phonons}} \approx J^Q_{\mathrm{edge}} = \frac{3\kappa_{2T}}{2}(T_m^2 - T_0^2). \tag{52}$$

Here, it is assumed that $J^Q_{\mathrm{phonons}} \propto T_m^5 - T_0^5$ can be neglected, which is the case for sufficiently low temperatures. (Alternatively, J^Q_{phonons} can be independently measured and accounted for.) The edge heat current J^Q_{edge} includes heat evacuated on all three connected arms. Supplementary Eq. (52) shows that κ_{2T} (in units of $\kappa_0 = \pi^2 k_B^2/3h$) can be extracted from a plot of P_{diss} vs $(T_m^2 - T_0^2)$: it is given by $2/(3\kappa_0)$ times the slope of the corresponding linear dependence. As explained in detail in the preceding Sections, T_m and T_0 are found from noise measurements [see Supplementary Eqs. (12), (46), and (48)]. The remaining ingredient for determining experimentally the heat conductance is the heat current J^Q_{edge} equal to the power P_{diss} dissipated at the central contact Ω_m. We explain now how the value of P_{diss} is found.

For states with only downstream heat flow (e.g., particle-like or integer edges), P_{diss} is simply equal to the injected power P [given by Supplementary Eq. (6)], $P_{\mathrm{diss}} = P$, since all injected energy is transported downstream to Ω_m (assuming small bulk losses)[35]. By contrast, for states with upstream heat flow, such as $\nu = 2/3$ and $\nu = 3/5$, part of the dissipated Joule heat in a hot spot may propagate upstream without heating the central contact. In this case, the power dissipated at Ω_m may be smaller than the total injected power[37], $P_{\mathrm{diss}} < P$. In order to use Supplementary Eq. (52) for the determination of κ_{2T}, it is thus important to explore what part of the injected power is actually used to heat the

contact Ω_m, i.e., to calculate the ratio P_{diss}/P. In the following, we perform this analysis in the regime of vanishing thermal equilibration.

The injected electrical power on each of the arms $i = 1, 2$ is $P_i = I^2/(2G_{2T})$. This power is dissipated not only inside Ω_m but also within a finite region of size l_{eq}^C outside Ω_m, i.e., at the hot spot (see Supplementary Figure 10). The power dissipated at this hot spot is[12]

$$P_{i,\text{h.s.}} = \frac{I^2}{2G_{2T}} \times \frac{\nu_-}{\nu_+}. \tag{53}$$

For $\nu_+ = 1$, which holds for all hole-conjugate states in the lowest Landau level (particularly for $\nu = 2/3$ and $\nu = 3/5$ considered in this work), we have $\nu_- = 1 - \nu$ and Supplementary Eq. (53) simplifies to

$$P_{i,\text{h.s.}} = \frac{I^2}{2G_{2T}}(1 - \nu). \tag{54}$$

In the absence of thermal equilibration, a fraction of this power propagates ballistically away from Ω_m (upstream), while the remaining part propagates to Ω_m and thus contributes to P_{diss}. In the extreme case where $P_{i,\text{h.s.}}$ is distributed uniformly over the edge modes, we have that only a part of $P_{i,\text{h.s.}}$ propagates downstream and heats Ω_m:

$$P_\Omega = \frac{n_d}{n_d + n_u} \times P_{i,\text{h.s.}}. \tag{55}$$

Here, n_d and n_u are the numbers of downstream and upstream edge channels, respectively. The remaining contribution, $\frac{n_u}{n_d+n_u} \times P_{i,\text{h.s.}}$ is instead carried by upstream propagating modes

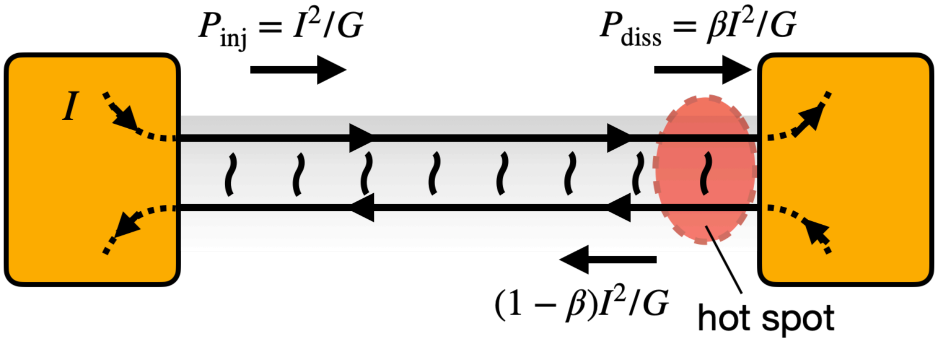

Supplementary Figure 10. **Dissipation of injected power.** Upon injecting a current in the left contact, only a fraction β of the injected power P_{diss} is dissipated in the right contact if the edge channels are not thermally equilibrated. The fraction $(1 - \beta)$ is carried upstream and does not heat the right contact.

away from Ω_m, i.e., back towards the source contact. The electrical power dissipated directly inside Ω_m is given by the voltage drop close to Ω_m. This directly dissipated power is given by

$$P_{\text{dir}} = \nu \frac{I^2}{2G_{2T}}. \tag{56}$$

Conservation of energy is ensured by $2P_{i,\text{h.s.}} + 2P_\Omega = P$. Adding the contributions (55) and (56), for arms 1 and 2, we obtain for the total dissipated power on Ω_m:

$$P_{\text{diss}} = 2P_{\text{dir}} + 2P_m = \frac{I^2}{G_{2T}}\left[\nu + \frac{n_d}{n_d + n_u}(1-\nu)\right] = \frac{I^2}{G_{2T}}\left[\frac{n_d + n_u \nu}{n_d + n_u}\right] \equiv \beta \frac{I^2}{G_{2T}}. \tag{57}$$

Supplementary Eq. (57) determines the fraction β of the injected power that is used to heat Ω_m in the absence of thermal equilibration. For $\nu = 2/3$ we have $n_u = n_d = 1$, which yields $\beta = 5/6 \approx 0.83$. For $\nu = 3/5$, with $n_d = 1$ and $n_u = 2$, we get $\beta = 11/15 \approx 0.73$. For an edge without counterpropagating modes ($n_u = 0$), we have $\beta = 1$, so that the dissipated power is equal to the injected power, as expected.

Using Supplementary Eq. (57), we can rewrite the power balance equation (52) in the form

$$\frac{I^2}{G_{2T}} = \frac{3\kappa_{2T}}{2\beta}(T_m^2 - T_0^2). \tag{58}$$

According to Supplementary Eq. (58), the heat conductance can be obtained by plotting P vs $(T_m^2 - T_0^2)$. The dimensionless heat conductance κ_{2T}/κ_0 is then given by $2\beta/(3\kappa_0)$ times the slope of the resulting linear dependence.

The calculation of β in Supplementary Eq. (57) is using a specific model of the contact and hot spot, and hence the actual value can differ somewhat. Also, let us emphasize that β is quite close to unity. Previous works[14,36] used $\beta = 1$ and obtained proper values for the equilibrated regime. In view of this, and since we do not have experimental control on β, we also choose $\beta = 1$ in our analysis (i.e., we assume that all dissipated power heats the central ohmic contact).

Supplementary Note 11. HEAT CONDUCTANCE: THEORY

In this Section, we discuss the expected value of the heat conductance κ_{2T}. The heat conductance for $\nu = 2/3$ was analyzed in various regimes in Supplementary Ref. 6, and we

briefly sketch the relevant results. As in the rest of this work, we focus on the regime of negligible thermal equilibration, $L \ll l_{\rm eq}$, where $l_{\rm eq}$ is the thermal equilibration length. As shown in Supplementary Ref. 6, this regime is subdivided into two. For very short systems (or very low temperatures), $L \ll L_T$, where L_T is the thermal length,

$$L_T = \frac{1}{(v_+^{-1} + v_-^{-1})T} \,, \tag{59}$$

the two-terminal heat conductance κ_{2T} is given (up to a small correction) by the number of edge modes,

$$\frac{\kappa_{2T}}{\kappa_0} = 2 \,, \qquad L \ll L_T \,. \tag{60}$$

For larger L (or higher T), the heat conductance is reduced due to partial back-scattering of thermal bosons at boundaries between the non-interacting contacts and interacting part of the wire, cf. Supplementary Note 7:

$$\frac{\kappa_{2T}}{\kappa_0} = 2 - \frac{4R}{1+R} = 2\sqrt{\frac{1}{7\Delta^2 - 4\sqrt{3\Delta^2\left(\Delta^2-1\right)} - 3}} \,, \qquad L_T \ll L \ll l_{\rm eq} \,. \tag{61}$$

In the non-interacting limit, $\Delta = 2$, we have $R = 0$ (no reflection of bosons at the interfaces), and Supplementary Eq. (61) yields $\kappa_{2T}/\kappa_0 = 2$. On the other hand, for $\Delta = 1$, corresponding to a regime of strong interaction, one finds

$$\frac{\kappa_{2T}}{\kappa_0} = 1 \,, \qquad L_T \ll L \ll l_{\rm eq} \,, \tag{62}$$

i.e., the thermal conductance us reduced by a factor of two. For the three-arm geometry of the present work, we obtain, for the regime of vanishing thermal equilibration and for strong interactions ($\Delta \approx 1$), a total heat conductance

$$3\frac{\kappa_{2T}}{\kappa_0} = 3. \tag{63}$$

The scattering analysis can be generalized to other hole-conjugated FQH edges (at fillings $\nu = p/(2p-1)$ with integer $p > 1$) with counter-propagating edge channels. The analysis is simplified at a low-energy fixed point where neutral modes are completely decoupled with a charge mode; this fixed point corresponds to $\Delta = 1$ for $\nu = 2/3$, discussed above. At such a fixed point, the neutral sector possesses a (global) $SU(p)$ symmetry[31,32]. Since this fixed point is a basin in a wide range of interaction-parameters space, it would be desirable to understand the value of thermal conductance at the fixed point.

We consider the case of $\nu = 3/5$ ($p = 3$) for a detailed computation. The edge of the $\nu = 3/5$ hosts one downstream mode ϕ_1 and two upstream modes $\phi_{1/3}$ and $\phi_{1/15}$, which satisfy the commutation relation $[\phi_1(x), \phi_1(x')] = i\pi\text{sgn}(x-x')$, $[\phi_{1/3}(x), \phi_{1/3}(x')] = -i\pi\text{sgn}(x-x')$, and $[\phi_{1/15}(x), \phi_{1/15}(x')] = -i\pi\text{sgn}(x-x')$, respectively; furthermore $[\phi_j(x), \phi_{j'}(x')] = 0$ for $j \neq j'$ with $j, j' = 1, 1/3, 1/15$. We define a downstream charge mode (ϕ_c) and two neutral modes (ϕ_{n_1} and ϕ_{n_2}) in terms of ϕ_1, $\phi_{1/3}$, and $\phi_{1/15}$ as

$$\begin{aligned}
\phi_c &= \sqrt{\frac{5}{3}}\left(\phi_1 + \frac{\phi_{1/3}}{\sqrt{3}} + \frac{\phi_{1/15}}{\sqrt{15}}\right), \\
\phi_{n_1} &= \frac{1}{\sqrt{2}}(\phi_1 + \sqrt{3}\phi_{1/3}), \\
\phi_{n_2} &= \frac{1}{\sqrt{6}}\left(\phi_1 + \frac{\phi_{1/3}}{\sqrt{3}} + \frac{10}{\sqrt{15}}\phi_{1/15}\right).
\end{aligned} \tag{64}$$

These charge and neutral modes are eigenmodes at the $SU(3)$ symmetric low-energy fixed point, and are thus decoupled.

We next calculate the thermal conductance employing the contact model depicted in Supplementary Figure 7. This is the model used for the noise calculation discussed in Supplementary Note 7. The edge segment consists of three regions: the left contact region, a central region, and the right contact region. While the bare modes ϕ_1, $\phi_{1/3}$, and $\phi_{1/15}$ are incoming modes out of the contacts or outgoing modes to the contacts, the charge and neutral modes are assumed to be eigenmodes in the central region. The left contact is thermally biased compared with the left contact: $T_L > T_R$. Scattering of the bosonic modes between different regions is described by the scattering matrices S_L and S_R, given as

$$\begin{pmatrix} \phi_c \\ \phi_{1/3,L} \\ \phi_{1/15,L} \end{pmatrix} = S_L \begin{pmatrix} \phi_{1,L} \\ \phi_{n_1} \\ \phi_{n_2} \end{pmatrix}, \qquad \begin{pmatrix} \phi_{1,R} \\ \phi_{n_1} \\ \phi_{n_2} \end{pmatrix} = S_R \begin{pmatrix} \phi_c \\ \phi_{1/3,R} \\ \phi_{1/15,R} \end{pmatrix}, \tag{65}$$

with

$$S_L = \begin{pmatrix} \sqrt{\frac{3}{5}} & \sqrt{\frac{3}{10}} & \sqrt{\frac{1}{10}} \\ -\sqrt{\frac{1}{3}} & \sqrt{\frac{2}{3}} & 0 \\ -\sqrt{\frac{1}{15}} & -\sqrt{\frac{1}{30}} & \frac{3}{\sqrt{10}} \end{pmatrix}, \qquad S_R = S_L^{-1}. \tag{66}$$

Here, $\phi_{j,L}$ and $\phi_{j,R}$ for $j = 1, 1/3, 1/15$ are incoming or outgoing modes in the left (L) and the right R contacts, respectively. This type of scattering problem is characterized

by transmission amplitude $\mathcal{T}(\omega)$ from the left to the right, calculated from the scattering matrices S_L and S_R as

$$\mathcal{T}(\omega) = \frac{T}{1 - Re^{2i\omega\tilde{\tau}}} = \frac{\nu}{1 - (1-\nu)e^{2i\omega\tilde{\tau}}} \tag{67}$$

with $T = \nu$ and $R = (1 - \nu)$ being the the transmission and reflection coefficients for the ϕ_1 mode. Furthermore, $\tilde{\tau} = L(1/v^+ + 1/v^-)/2$ is the mean flight time through the central region, v_+ and v_- are the velocities of the charge mode and the neutral modes, respectively. Note that the $SU(3)$ symmetry of the neutral sector renders the velocities of the two neutral modes to be the same. Calculating the energy current in the edge mode ϕ_1, we obtain the reflected thermal conductance κ_{12} with

$$\kappa_{12} = \kappa_0 \left(1 - \frac{6}{\pi^2 \bar{T}^2} \int_0^\infty \frac{\omega d\omega}{e^{\omega/\bar{T}} - 1} |\mathcal{T}(\omega)|^2 \right). \tag{68}$$

Here, $\bar{T}$ is the average temperature $\bar{T} = (T_L + T_R)/2$. For very short length $L \ll L_T \equiv 1/[(v_+^{-1} + v_-^{-1})\bar{T}]$, κ_{12} becomes zero, and hence the two-terminal heat conductance $\kappa_{2T} = (n_u + n_d)\kappa_0 - 2\kappa_{12}$ is given by the total number of modes $\kappa_{2T} = (n_u + n_d)\kappa_0$. On the other hand, in the regime of $L_T \ll L \ll l_{\text{eq}}$, we have $\kappa_{12} = \kappa_0 (2R/(1+R))$ and thus

$$\kappa_{2T} = \kappa_0 \left(n_u + n_d - \frac{4R}{1+R}\right) = \kappa_0 \left(n_u + n_d - \frac{4(1-\nu)}{2-\nu}\right). \tag{69}$$

Note that Supplementary Eqs. (67)-(69) generically holds for any hole-conjugated states with $\nu = p/(2p-1)$ at a $SU(p)$ fixed point. The case $p = 2$ corresponds to $\nu = 2/3$, with $n_u = n_d = 1$. Supplementary Eq. (69) yields in this case $\kappa_{2T} = \kappa_0$, in agreement with Supplementary Eq. (62). For $p = 3$ we have $\nu = 3/5$, with $n_u = 2$ and $n_d = 1$, and Supplementary Eq. (69) yields

$$\frac{\kappa_{2T}}{\kappa_0} = 13/7 \approx 1.86, \tag{70}$$

as stated in the main text. The total heat conductance of the three-armed devices is then obtained as $3\kappa_{2T}/\kappa_0 \approx 5.57$.

Supplementary Note 12. EXTRACTING HEAT CONDUCTANCE AND TEMPERATURE DEPENDENCE OF UPSTREAM NOISE FROM EXPERIMENTAL DATA AND COMPARISON TO THEORY

In this Section, we present details of determining the heat conductance and the temperature dependence of the upstream noise from the experimental data by using the theoretical

framework developed in Supplementary Note 6-Supplementary Note 11. Further, we compare the obtained results for the upstream noise and the heat conductance to the theoretical predictions for the FQH state with filling factor $\nu = 2/3$.

A. Determining the central-contact temperature T_m

As a first step, we extract the central contact temperature T_m for a given injected current I (or equivalently a given injected power P). For this purpose, we combine Supplementary Eq. (12) for the downstream excess noise $S^{\mathrm{D}}_{\mathrm{excess}}$ with the microscopically computed $\overline{(\Delta I_m)^2}$ from Supplementary Eqs. (46) and (48). We further use the approximation (47). As a result, we obtain the following equation relating $\Delta T = T_m - T_0$ and the measured noises:

$$\frac{4\alpha}{3} \times G_{2T} k_B \Delta T \approx S^{D}_{\mathrm{excess}} - \frac{1}{9}\left[S^{S_1}_{\mathrm{excess}} + S^{S_2}_{\mathrm{excess}} + S^{U}_{\mathrm{excess}}\right], \quad G_{2T} = \frac{\nu e^2}{h}. \tag{71}$$

Here, $\alpha = (4\nu_+ - 3\nu_-)/4\nu_+$, which yields $\alpha = 3/4$ for $\nu = 2/3$ and $\alpha = 7/10$ for $\nu = 3/5$. Defined in this way, α is a parameter describing the deviation from the Nyquist-Johnsson relation (13), independently of the device geometry. Furthermore, $S^{\mathrm{D}}_{\mathrm{excess}}$ and $S^{\mathrm{U}}_{\mathrm{excess}}$ are measured directly for various lengths between Ω_m and the upstream contact U with the amplifier $\mathrm{A_U}$ (see Supplementary Figure 6). By contrast, the noise from sources, $S^{S_1}_{\mathrm{excess}}$ and $S^{S_2}_{\mathrm{excess}}$, are not directly measured. However, since these noises are of exactly the same nature as the upstream noise $S^{\mathrm{U}}_{\mathrm{excess}}$, they should be essentially equal to $S^{\mathrm{U}}_{\mathrm{excess}}$ at lengths equal to the distances between the sources and Ω_m. In the experiment, these lengths were fixed at 30μm and 150μm for S_1 and S_2, respectively. Thus, all the terms in the r.h.s. of Supplementary Eq. (71) are obtained from experimental measurements, which allows us to determine ΔT as a function of I.

B. Determination of temperature dependence of upstream noise. Comparison to theory.

Next, we plot the measured $S^{\mathrm{U}}_{\mathrm{excess}}$ against ΔT extracted from Supplementary Eq. (71) as explained in Supplementary Note 12 A. The results are shown in Supplementary Figure 11 for three different values of the length between Ω_m and the upstream contact U. While the noise decreases with increasing length, we see that this dependence is rather weak. This

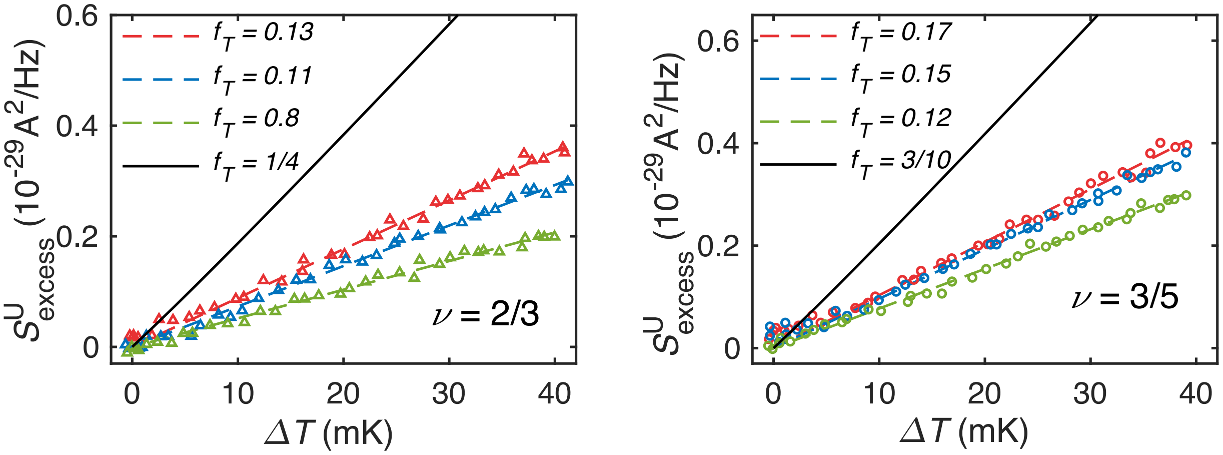

(a) (b)

Supplementary Figure 11. **Extraction of upstream noise.** Upstream excess noise $S^{\mathrm{U}}_{\mathrm{excess}}$ vs $\Delta T = T_m - T_0$, where T_m is the temperature of the hot central contact and T_0 is the base temperature. (a) Filling $\nu = 2/3$. Triangle symbols: experimentally measured noise, with the temperature ΔT determined as explained in Supplementary Note 12 B, for three different lengths for the upstream heat propagation: 15μm (red), 45μm (blue), and 75μm (green). Dashed lines are linear fits, which gives thermal Fano factors $f_T \equiv S^{\mathrm{U}}_{\mathrm{excess}}/(2G_{2T}k_B\Delta T)$. The black solid line is the theoretical result (for vanishing thermal equilibration and under assumption of no losses to the bulk), $f_T = 1/4$ [see Supplementary Eq. (72)], which becomes $S^{\mathrm{U}}_{\mathrm{excess}} \approx 0.017\Delta T$ when the noise is measured in units of $10^{-29}\mathrm{A}^2/\mathrm{Hz}$ and the temperature in mK. (b) Filling $\nu = 3/5$. Circles denote measured data with color coding as that in (a). The black line is the theoretical result in Supplementary Eq. (73): $f_T = 3/10$ or $S^{\mathrm{U}}_{\mathrm{excess}} \approx 0.019\Delta T$ in the units referred to in (a).

is particularly true for shorter systems: the noise decreases only by $\approx 10\%$ when the length increases from 15μm to 45μm. This is a clear demonstration of the fact that the system is in the regime with essentially vanishing heat equilibration: the heat propagates ballistically from the central contact Ω_m to the noise spot near the contact U. A slow reduction of noise with increasing length may be due to two reasons: (i) weak thermal equilibration, and (ii) losses of heat propagating along the edge to the environment ("bulk"), including phonons as well as electronic modes in the bulk mediated by Coulomb interaction[38]. As the data on heat conductance demonstrate (see Supplementary Note 12 C below), the dominant source in our experiment is losses to the bulk, while the effect of thermal equilibration within the

edge is negligible.

In Supplementary Figure 11 we further compare the results for $S^{\rm U}_{\rm excess}(\Delta T)$ (obtained from experimental measurements as detailed above) to the theoretical formula for the upstream noise in the regime of vanishing thermal equilibration,

$$S^{\rm U}_{\rm excess} = \frac{1}{3}\frac{e^2}{h}k_B\Delta T \equiv \frac{1}{4}\times 2G_{2T}k_B\Delta T, \qquad G_{2T} = \frac{2e^2}{3h}\,. \tag{72}$$

$$S^{\rm U}_{\rm excess} = \frac{9}{25}\frac{e^2}{h}k_B\Delta T \equiv \frac{3}{10}\times 2G_{2T}k_B\Delta T, \qquad G_{2T} = \frac{3e^2}{5h}\,. \tag{73}$$

Equation (72) is obtained from the general expression (15) of $S^{\rm U}_{\rm excess}$ by inserting Supplementary Eq. (49) and using the approximation (50). Similarly, Supplementary Eq. (73) follows by using Supplementary Eqs. (50) and (51) in Supplementary Eq. (15). Supplementary Equations (72) and (73) yield the excess upstream noise in the limit of zero thermal equilibration and no losses to the bulk, i.e., when the heat from Ω_m propagates ballistically and without losses to the noise spot near the contact U (see Supplementary Figure 6).

Supplementary Eqs. (72)-(73) define the thermal Fano factors $f_T \equiv S^{\rm U}_{\rm excess}/(2G_{2T}k_B\Delta T)$, which for $\nu = 2/3$ and $\nu = 3/5$ gives $f_T = 1/4$ and $f_T = 3/10$ respectively.

We see that the linear dependencies of $S^{\rm U}_{\rm excess}(\Delta T)$ is in full consistency with experimental data. At the same time, the experimental value of the noise for the shortest distance (for which the effect of losses and thermal relaxations are negligible as pointed out above) is approximately twice smaller than the theoretical prediction. It is not clear to us at present what is the source of this discrepancy.

C. Determination of heat conductance. Comparison to theory.

To extract the heat conductance κ_{2T}/κ_0 from the experimental measurements, we use the approach described in Supplementary Note 10. In accordance with the power balance equation (58), we plot in Supplementary Figure 12 the injected power P as a function of $T_m^2 - T_0^2 = \Delta T^2 + 2T_0\Delta T$. Here, the base temperatures $T_0 = 14$mK for $\nu = 2/3$ and $T_0 = 11$mK for $\nu = 3/5$ are extracted from equilibrium noise (i.e., noise at zero current bias $I = 0$). The temperature difference ΔT is obtained from the experimentally measured noise according to Supplementary Eq. (71). We extract the heat conductance for three lengths, 15μm (plotted in red), 45μm (blue), and 85μm (green), between Ω_m and the upstream contact U. The heat conductance κ_{2T}/κ_0 is obtained as $2/\kappa_0$ times the slope of the resulting

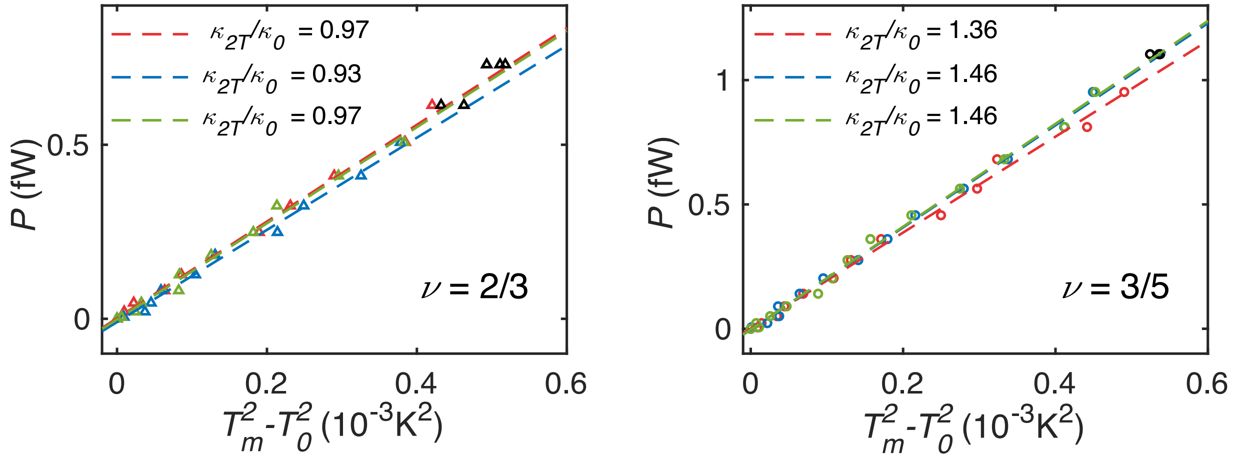

(a) (b)

Supplementary Figure 12. **Extraction of the thermal conductance for fractional states.** Injected power P vs $T_m^2 - T_0^2$. (a) Filling $\nu = 2/3$. Triangles denote measured data for three lengths between the central contact Ω_m and the upstream contact U: 15μm (red), 45μm (blue), and 75μm (green). The heat conductance κ_{2T}/κ_0 is given by the slope of the dependence P vs $T_m^2 - T_0^2$, multiplied by $2/(3\kappa_0)$, [see Supplementary Eq. (57), were we take $\beta = 1$.]. This results in $\kappa_{2T}/\kappa_0 \approx 0.97$, 0.93, and 0.97 for the lengths 15μm, 45μm, and 75μm, respectively. (b) Filling $\nu = 3/5$. Circles denote measured data at the same lengths as in (a). We find $\kappa_{2T}/\kappa_0 \approx 1.36$, 1.46, and 1.46 for the lengths 15μm, 45μm, and 75μm, respectively.

linear dependences, see Supplementary Eq. (58) (as motivated in Supplementary Note 10, we have taken $\beta = 1$ for all considered states). The slopes are extracted in the linear regime $0 \lesssim T_m^2 - T_0^2 \lesssim 0.4 \times 10^{-3}\mathrm{K}^2$. For $\nu = 2/3$, we find $\kappa_{2T}/\kappa_0 \approx 0.97$, 0.93, and 0.97 for the lengths 15μm, 45μm, and 75μm, respectively. For $\nu = 3/5$, we find $\kappa_{2T}/\kappa_0 \approx 1.36$, 1.46, and 1.46 for the lengths 15μm, 45μm, and 75μm, respectively. The result is that κ_{2T} independent on the length, within the uncertainty determined by the statistical scattering of data.

The length-independence of the measured heat conductance is a strong evidence of the absence (within the accuracy of our data) of heat equilibration at the length scales studied in our experiment. Indeed, in the presence of heat equilibration, the heat conductance κ_{2T} of a $\nu = 2/3$ edge shows, with increasing length L, a crossover from the ballistic behavior (L-independent κ_{2T}) at $L \ll l_{\mathrm{eq}}$ to the diffusive behavior, $\kappa_{2T} \propto 1/L$ at $L \gg l_{\mathrm{eq}}$, see Supplementary Ref. 6. Our results thus imply that the devices studied in this experiment

are in the regime $L \ll l_{\rm eq}$.

Let us further comment on the insensitivity of the thermal-conductance measurements to losses to the bulk. Indeed, for the length 75μm, these losses are clearly observable in the upstream noise measurement, see Supplementary Figure 11. At the same time, the results for the thermal conductance shown in Supplementary Figure 12 do not show any trace of the losses. The reason for this insensitivity of the heat conductance measured with the present method to bulk losses is as follows[37]. The measurement protocol matches the incoming and outgoing heat flows with respect to Ω_m [see Supplementary Eq. (58)]. The crucial point is that the heat is evacuated from the central contact Ω_m via the edge states. Whether the full outgoing energy reaches another electrode or some part of it leaks to the bulk on the way there is irrelevant. The assumption here is that the heat that is leaking to the bulk does not return to the central contact Ω_m, which is expected to be a very good approximation. This should be contrasted to the effect of heat equilibration within the edge, which leads to back-scattering of heat that thus returns to Ω_m, leading to a decrease of κ_{2T}.

Summarizing, our results for the upstream noise and the conductance are interpreted in the following way: (i) the thermal relaxation within the edge is negligible at studied length scales, and (ii) leakage of energy to the bulk leads to reduction of the upstream noise with distance but is irrelevant for the thermal conductance.

Finally, we compare the value $\kappa_{2T}/\kappa_0 \approx 1$ obtained from experimental data at $\nu = 2/3$ with the theoretical result (62) derived for the thermally non-equilibrated regime and strong interaction ($\Delta \approx 1$). We see that the experimental value is in good agreement with the theoretical prediction. For $\nu = 3/5$, the obtained $\kappa_{2T}/\kappa_0 \approx 1.45$ lies below the predicted $\kappa_{2T}/\kappa_0 = 13/7 \approx 1.86$ [see Supplementary Eq. (70)]. This deviation could be related to a deviation of the system from the infrared fixed point.

SUPPLEMENTARY REFERENCES

---

[1] J. B. Johnson, Phys. Rev. **32**, 97 (1928).

[2] H. Nyquist, Phys. Rev. **32**, 110 (1928).

[3] E. Sivre, A. Anthore, F. D. Parmentier, A. Cavanna, U. Gennser, A. Ouerghi, Y. Jin, and F. Pierre, Nature Physics **14**, 145 (2018).

[4] A. O. Slobodeniuk, I. P. Levkivskyi, and E. V. Sukhorukov, Phys. Rev. B **88**, 165307 (2013).

[5] A. Aharon-Steinberg, Y. Oreg, and A. Stern, Phys. Rev. B **99**, 041302 (2019).

[6] I. Protopopov, Y. Gefen, and A. Mirlin, Annals of Physics **385**, 287 (2017).

[7] Y. Cohen, Y. Ronen, W. Yang, D. Banitt, J. Park, M. Heiblum, A. D. Mirlin, Y. Gefen, and V. Umansky, Nature Communications **10**, 1920 (2019).

[8] F. Lafont, A. Rosenblatt, M. Heiblum, and V. Umansky, Science **363**, 54 (2019).

[9] C. Nosiglia, J. Park, B. Rosenow, and Y. Gefen, Phys. Rev. B **98**, 115408 (2018).

[10] J. Park, A. D. Mirlin, B. Rosenow, and Y. Gefen, Phys. Rev. B **99**, 161302 (2019).

[11] C. Spånslätt, J. Park, Y. Gefen, and A. D. Mirlin, Phys. Rev. Lett. **123**, 137701 (2019).

[12] C. Spånslätt, J. Park, Y. Gefen, and A. D. Mirlin, Phys. Rev. B **101**, 075308 (2020).

[13] J. Park, C. Spånslätt, Y. Gefen, and A. D. Mirlin, Phys. Rev. Lett. **125**, 157702 (2020).

[14] M. Banerjee, M. Heiblum, A. Rosenblatt, Y. Oreg, D. E. Feldman, A. Stern, and V. Umansky, Nature **545**, 75 EP (2017).

[15] S. K. Srivastav, R. Kumar, C. Spånslätt, K. Watanabe, T. Taniguchi, A. D. Mirlin, Y. Gefen, and A. Das, Phys. Rev. Lett. **126**, 216803 (2021).

[16] C. L. Kane, M. P. A. Fisher, and J. Polchinski, Phys. Rev. Lett. **72**, 4129 (1994).

[17] C. Spånslätt, Y. Gefen, I. V. Gornyi, and D. G. Polyakov, Phys. Rev. B **104**, 115416 (2021).

[18] E. V. Sukhorukov and D. Loss, Phys. Rev. B **59**, 13054 (1999).

[19] A. Mu and D. Segal, arXiv preprint arXiv:1902.06312 (2019).

[20] E. Zhitlukhina, M. Belogolovskii, and P. Seidel, Applied Nanoscience **10**, 5121 (2020).

[21] J. Rech, T. Jonckheere, B. Grémaud, and T. Martin, Phys. Rev. Lett. **125**, 086801 (2020).

[22] M. Hasegawa and K. Saito, Phys. Rev. B **103**, 045409 (2021).

[23] J. Eriksson, M. Acciai, L. Tesser, and J. Splettstoesser, Phys. Rev. Lett. **127**, 136801 (2021).

[24] A. Popoff, J. Rech, T. Jonckheere, L. Raymond, B. Grémaud, S. Malherbe, and T. Martin, arXiv preprint arXiv:2106.05679 (2021).

[25] O. S. Lumbroso, L. Simine, A. Nitzan, D. Segal, and O. Tal, Nature **562**, 240 (2018).

[26] E. Sivre, H. Duprez, A. Anthore, A. Aassime, F. D. Parmentier, A. Cavanna, A. Ouerghi, U. Gennser, and F. Pierre, Nature Communications **10**, 5638 (2019).

[27] S. Larocque, E. Pinsolle, C. Lupien, and B. Reulet, Phys. Rev. Lett. **125**, 106801 (2020).

[28] A. Rosenblatt, S. Konyzheva, F. Lafont, N. Schiller, J. Park, K. Snizhko, M. Heiblum, Y. Oreg, and V. Umansky, Phys. Rev. Lett. **125**, 256803 (2020).

[29] A. M. Chang, Rev. Mod. Phys. **75**, 1449 (2003).

[30] D. B. Gutman, Y. Gefen, and A. D. Mirlin, Phys. Rev. B **80**, 045106 (2009).

[31] C. L. Kane and M. P. A. Fisher, Phys. Rev. B **51**, 13449 (1995).

[32] J. E. Moore and X.-G. Wen, Phys. Rev. B **57**, 10138 (1998).

[33] J. K. Jain, Phys. Rev. Lett. **63**, 199 (1989).

[34] R. Kumar, S. K. Srivastav, C. Spånslätt, K. Watanabe, T. Taniguchi, Y. Gefen, A. D. Mirlin, and A. Das, arXiv preprint arXiv:2107.12616 (2021), https://arxiv.org/abs/2107.12616.

[35] S. Jezouin, F. D. Parmentier, A. Anthore, U. Gennser, A. Cavanna, Y. Jin, and F. Pierre, Science **342**, 601 (2013).

[36] M. Banerjee, M. Heiblum, V. Umansky, D. E. Feldman, Y. Oreg, and A. Stern, Nature **559**, 205 (2018).

[37] K. K. W. Ma and D. E. Feldman, Phys. Rev. B **99**, 085309 (2019).

[38] D. B. Gutman, I. V. Protopopov, A. L. Burin, I. V. Gornyi, R. A. Santos, and A. D. Mirlin, Phys. Rev. B **93**, 245427 (2016).
